# Genomic Mechanisms Accounting for the Adaptation to Parasitism in Nematode-Trapping Fungi

**DOI:** 10.1371/journal.pgen.1003909

**Published:** 2013-11-14

**Authors:** Tejashwari Meerupati, Karl-Magnus Andersson, Eva Friman, Dharmendra Kumar, Anders Tunlid, Dag Ahrén

**Affiliations:** 1 Microbial Ecology Group, Department of Biology, Lund University, Ecology Building, Lund, Sweden; 2 Department of Genetics and Plant Breeding, N.D. University of Agriculture and Technology, Faizabad, India; 3 BILS Bioinformatics Infrastructure for Life Sciences, Department of Biology, Lund University, Ecology Building, Lund, Sweden; MicroTrek Incorporated, United States of America

## Abstract

Orbiliomycetes is one of the earliest diverging branches of the filamentous ascomycetes. The class contains nematode-trapping fungi that form unique infection structures, called traps, to capture and kill free-living nematodes. The traps have evolved differently along several lineages and include adhesive traps (knobs, nets or branches) and constricting rings. We show, by genome sequencing of the knob-forming species *Monacrosporium haptotylum* and comparison with the net-forming species *Arthrobotrys oligospora*, that two genomic mechanisms are likely to have been important for the adaptation to parasitism in these fungi. Firstly, the expansion of protein domain families and the large number of species-specific genes indicated that gene duplication followed by functional diversification had a major role in the evolution of the nematode-trapping fungi. Gene expression indicated that many of these genes are important for pathogenicity. Secondly, gene expression of orthologs between the two fungi during infection indicated that differential regulation was an important mechanism for the evolution of parasitism in nematode-trapping fungi. Many of the highly expressed and highly upregulated *M. haptotylum* transcripts during the early stages of nematode infection were species-specific and encoded small secreted proteins (SSPs) that were affected by repeat-induced point mutations (RIP). An active RIP mechanism was revealed by lack of repeats, dinucleotide bias in repeats and genes, low proportion of recent gene duplicates, and reduction of recent gene family expansions. The high expression and rapid divergence of SSPs indicate a striking similarity in the infection mechanisms of nematode-trapping fungi and plant and insect pathogens from the crown groups of the filamentous ascomycetes (Pezizomycotina). The patterns of gene family expansions in the nematode-trapping fungi were more similar to plant pathogens than to insect and animal pathogens. The observation of RIP activity in the Orbiliomycetes suggested that this mechanism was present early in the evolution of the filamentous ascomycetes.

## Author Summary

Nematode-trapping fungi are a group of soil-living fungi found worldwide. They capture and kill nematodes and are used as biocontrol agents against parasitic nematodes. The infection structures differ morphologically. Their traps can be classified into several main groups, including adhesive knobs, adhesive nets, adhesive branches and constricting rings. We have sequenced the genome of *Monacrosporium haptotylum*, a knob-forming fungus, and compared it with the genome of *Arthrobotrys oligospora*, a net-forming fungus. Comparative and functional genomics analyses of the predicted functions of the two species' genes provide new insights into how the nematode-trapping lifestyle has evolved. Two genomic mechanisms are likely to have been responsible for the adaptation to this lifestyle: the formation of new genes through gene duplications, including gene family expansions, and the differential gene expression of orthologous genes in the two fungi. We identified a large number of genes that were found only in these two species (either shared by both or found just in one or other genome); many of these were highly expressed and differentially regulated during nematode infection. Our results suggest that the function and expression patterns of these genes have evolved in response to interactions with nematode hosts.

## Introduction

Ascomycota is the largest phylum of kingdom Fungi and includes approximately 33,000 described species [1]. The phylum is divided into three monophyletic subphyla: Taphrinomycotina, Saccharomycotina and Pezizomycotina [2]. Pezizomycotina is the largest subphylum and includes the vast majority of filamentous, fruit-body-producing species. Molecular phylogeny resolves Orbiliomycetes and Pezizomycetes as the early-diverging lineages of the Pezizomycotina, with the remaining seven classes sampled forming a well-supported crown clade [2]. The Orbiliomycetes consists of a single order (Orbiliales) and one family (Orbiliaceae). This family is best known for containing nematode-trapping fungi [3]. These soil-living fungi capture and kill nematodes using specialized infection structures [4], [5], which are morphologically distinct structures called traps. The remarkable morphological adaptations and the dramatic infection process of the nematode-trapping fungi have fascinated mycologists for centuries. Another reason for the interest in the nematode-trapping fungi has been their ability to act as biocontrol agents against parasitic nematodes [5].

**Figure 1:**
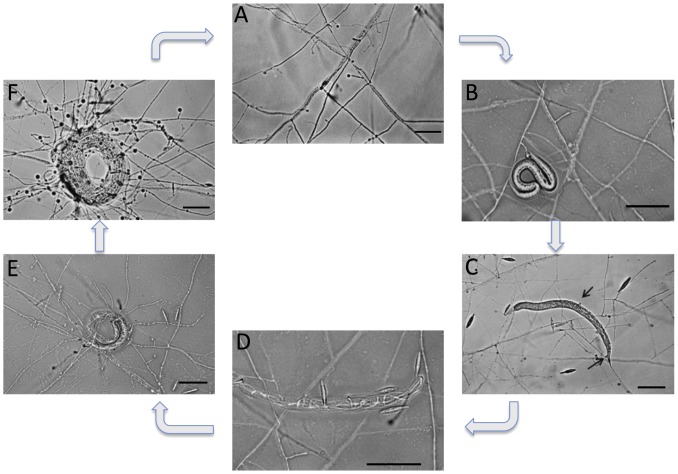
Life cycle of the nematode-trapping fungus *M. haptotylum*. (A) Saprophytic growth with hyphae and knob; (B) Newly captured nematode; (C) Knobs (black arrows) adhering to immobilized nematode (∼1 hour after adding the nematode); (D) Fungal degradation of the nematode; (E) Emergence of new hyphae from the nematode; (F) Trap formation after degrading nematode. The whole infection process from adhesion to new trap formation after degrading the nematode lasts ∼36 hours. Scale bar, 50 µm. The infected nematode shown is *C. briggsae*.

The morphology of the nematode trap differs depending on the species, and the major types group according to molecular phylogeny data [6]–[8]. The traps can be divided into four major types: adhesive nets, adhesive knobs, adhesive branches and constricting rings [9]. Species that form constricting rings are monophyletic and found near the base of the tree of nematode-trapping fungi. Among the species that form adhesive traps, those that form adhesive knobs and adhesive nets are a sister clade separated from those that form adhesive branches [6]. Species with adhesive traps capture nematodes using extracellular polymers that accumulate at the site of infection [10], whereas those with constricting rings ensnare the nematode by rapid swelling of the ring cells. In both adhesive and constricting-ring types, the cuticles of the captured nematode are penetrated and an infection bulb is formed inside the nematode. At the time of penetration, the nematode is paralyzed. Subsequently, the nematode is killed, fungal hyphae grow inside it, and fungal enzymes degrade its tissues. Finally, the nutrients are taken up and translocated to new mycelia that grow out from the digested nematode [11] (Figure 1). Although the morphology of traps varies extensively, the nematode-trapping fungi are generalists and they can infect many different nematode species [5].

Over the last few years, comparative analyses of genome sequences have provided novel insights into the evolution of the diverse lifestyles of the filamentous ascomycetes. The species include those feeding on dead organic matter only (saprophytes) [12]–[14], plant pathogens [15], [16], human pathogens [17], [18] and insect pathogens [19]. All these species belong to the crown clades of the Pezizomycotina. Recently, the first genome of a nematode-trapping fungus of the Orbiliomycetes was sequenced [20]. The species, *Arthrobotrys oligospora* forms adhesive networks. Comparative genomics of *A. oligospora* with 10 other fungal genomes revealed several genes that were shared specifically between *A. oligospora* and pathogenic fungi. In addition, large gene families related to pathogenicity were identified in the *A. oligospora* genome, including the subtilisin, cellulase, cellobiohydrolase and pectin esterase families [20]. In addition, the regulation of proteins during trap formation was studied. A majority of the upregulated proteins were classified as being involved in translation, posttranslational modification, amino acid metabolism, carbohydrate metabolism, energy conversion, cell wall and membrane biogenesis [20].

To gain further insights into the evolution of parasitism in the nematode-trapping fungi of the Orbiliomycetes, in this study we have sequenced the genome of a knob-forming species, *Monacrosporium haptotylum*. A recent study using five protein-coding genes estimated the split between species that form adhesive nets and those that form adhesive knobs to have occurred 198–208 million years ago [21]. However, the taxonomic assignment of the fossils and the identification of traps has been questioned [22] and therefore the evolutionary history of the nematode-trapping fungi remains uncertain. Together with *A. oligospora*, *M. haptotylum* is the nematode-trapping fungus in which the infection mechanism has been studied in most detail [23]. A major advantage of using *M. haptotylum* in infection experiments is that the trap cells (knobs) can be isolated from a mycelium growing in liquid cultures [24]. The isolated traps are functionally intact, that is, they can capture and kill nematodes, including *Caenorhabditis briggsae* (Figure 1). Accordingly, the system provides unique opportunities to identify genes that are differentially expressed in the trap cells and in the fungus during the various stages of infection [25], [26].

Our comparative genomics studies of *M. haptotylum* and *A. oligospora* showed that two genomic mechanisms are likely to be involved in the adaptation to pathogenicity in nematode-trapping fungi of the Orbiliomycetes. Firstly, gene duplications including gene family expansions indicate that the formation of novel genes was an important mechanism during the evolution of parasitism. Secondly, gene expression of orthologs indicates that differential gene expression between the two nematode-trapping fungi was another important adaptation to parasitism. Lineage- and species-specific genes are significantly shorter than genes shared by other fungi (core genes). Many of these short genes encode secretory proteins and are small secreted proteins (SSPs), which are likely to contain proteins that directly interact with the host during infection. The SSPs have undergone rapid divergence and many are orphans, that is, they lack known homologs and do not contain any Pfam domains. Expression of many of the SSPs was highly upregulated and expressed during nematode infection. We propose that the evolution of the infection-expressed SSPs involved tandem duplications followed by a rapid divergence governed by a repeat-induced point mutation (RIP) mechanism. The RIP mechanism has been demonstrated to play a central role in the genome evolution of several ascomycetes in the crown clades of the Pezizomycotina [12], [15], [19], [27]. In addition, RIP has been identified in *A. oligospora* [20] and *M. haptotylum* (this study), both belonging to the Orbiliomycetes. The presence of a RIP mechanism in the Orbiliomycetes suggests that RIP was present early in the evolution of the filamentous ascomycetes (Pezizomycotina). The rapid divergence of SSPs coupled with differential expression of these genes indicates a striking similarity in the evolution of the infection mechanisms of the nematode-trapping fungi from the basal lineages and of plant pathogenic and insect pathogenic fungi from the crown clades of the filamentous ascomycetes [15], [16], [19]. An analysis of the expansion of gene families along the lineages of pathogenic fungi of the Pezizomycotina revealed that patterns of expansion in the nematode-trapping fungi are most similar to that in plant pathogenic fungi.

## Results

### Genome sequencing and general features of the *M. haptotylum* genome

The genome of *M. haptotylum* was sequenced to 28× coverage by 454 pyrosequencing (Table S1). Based on these data, the genome size of *M. haptotylum* was estimated to be 40.4 Mb and the number of protein-coding genes to be 10,959. The completeness of the sequenced genome and prediction of open reading frames were validated by analyses of transcriptome sequences. In total, 99% of the 422,883 pyrosequencing reads and 83–99% of previously generated expressed sequence tag (EST) sequences [25], [26] were unambiguously mapped to the genome (Table S2). The genome assembly is therefore likely to cover the vast majority of the genes in the genome of *M. haptotylum*. Furthermore, analyses of RNASeq data showed that almost all of the predicted protein-coding genes (10,899 out of 10,959) were expressed by the fungus either in the saprophytic or the parasitic stage.

The estimated genome size and number of protein-coding genes in *M. haptotylum* are almost identical to the equivalent numbers for *A. oligospora* (Table 1) and similar to those of other ascomycetes (Table S3). In total, 149 putative tRNA genes were identified in the genome of *M. haptotylum* (Table S4), which is similar to the number in *A. oligospora* (Table 1), in insect pathogenic fungi [19] and in several other fungi (http://lowelab.ucsc.edu/GtRNAdb). The distribution of proteins into different EuKaryotic Orthologous Groups (KOG) categories was similar in *M. haptotylum* and *A. oligospora* (Figure S1). The *M. haptotylum* genome contained 271 genes per Mb and the average number of exons per gene was 3.3, which is similar to that of the *A. oligospora* genome.

**Table 1:** Main features of the *M. haptotylum* and *A. oligospora* genomes.

Features	*M. haptotylum*	*A. oligospora*
Size (Mb)	40.4	40.1
Coverage	28×	37×
GC content (%)	45.24	44.45
Protein-coding genes	10,959	11,479
Gene density (genes per Mbp)	271	286
Exons per gene	3.3	3.8
Average length of introns (bp)	108	90
Total number of tRNA genes	149	154
Secreted proteins	1,666	1,568
Proteins with Pfam domain	7,455	7,555

Transposable elements (TEs) were identified in *M. haptotylum* and *A. oligospora* and annotated as retrotransposons (Class I) or DNA transposons (Class II) (Table S5). The genome of *M. haptotylum* contains approximately twice as many TEs as the genome of *A. oligospora*. In both organisms two-thirds of the TEs belong to Class I. For most TEs the numbers located in the genes were similar in *M. haptotylum* and *A. oligospora*. The mariner and mariner ant1 TE families were more abundant in the genes of *M. haptotylum* than in those of *A. oligospora* (Figure S2).

Duplicated genes were identified based on ortholog family assignment using orthoMCL [28]. Tandem duplications of the genes in families were identified in *M. haptotylum*: two or more genes adjacent in the genome belonging to the same family were considered to be tandem duplicated. In total, 147 duplicated pairs consisting of 272 genes (2.5% of all genes) were identified as being located in tandem positions. A permutation test (1,000 permutations) was performed by random reordering of the genes in the genome; this showed that tandemly duplicated genes were significantly more common than expected by chance (P<0.001). Only a few of the tandemly duplicated genes, 6 out of 147, were located near (<10 kb) a transposon, indicating that the duplications were not a consequence of transposon activity.

The proportion of genes encoding secreted proteins in *M. haptotylum* was estimated to be 15.2% (1,666 proteins), which is similar to that predicted in *A. oligospora* (Table 1). The proportions of secreted proteins in the two nematode-trapping fungi was comparable to that predicted for the insect pathogen *Metarhizium anisopliae* (13.2%; 1,394 proteins) but higher than in the saprotroph *Neurospora crassa* (10.58%; 1,042 proteins) and in the animal pathogen *Aspergillus fumigatus* (10.3%; 996 proteins).

A BLAST analysis was conducted against the pathogen–host interaction protein database (PHI-base) [29], which contain a collection of experimentally verified pathogenicity, virulence and effector genes from fungi, oomycetes and bacteria and enables computational identification of candidate pathogenicity genes. We identified 1,161 proteins in *M. haptotylum* (10.6% of all genes) and 1,132 proteins in *A. oligospora* (9.9%) that are similar to proteins in the PHI-base database, particularly from plant pathogens. The most abundant PHI-base genes in *M. haptotylum* and *A. oligospora* encoded transporters and proteins involved in signaling, oxidation, transcription regulation and metabolism (Table S6). The putative PHI-base proteins of *M. haptotylum* contained 555 Pfam domains, of which the most common are shown in Table S7.

### Phylogeny

**Figure 2:**
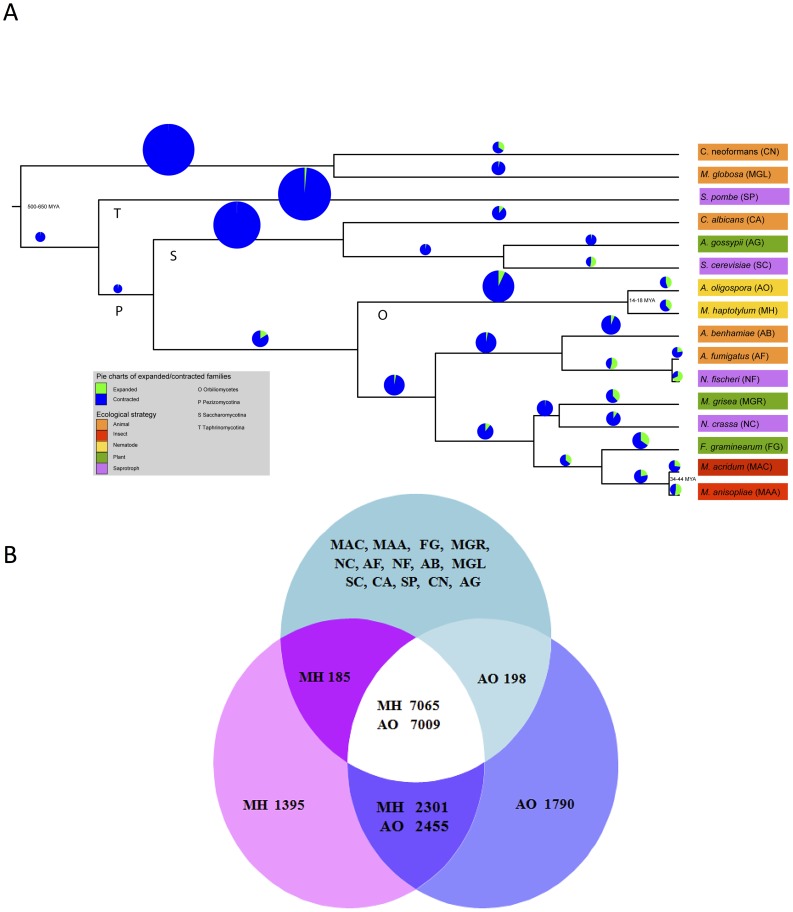
Gene family expansions, contractions and lineage-specific proteins of the nematode-trapping fungi *M. haptotylum* and *A. oligospora*. (A) Rooted maximum likelihood tree constructed from 602 single copy orthologous proteins using the Dayhoff amino acid substitution model showing evolutionary relationships of 16 fungal species. Branches labeled with letters show the taxonomic classification O: Orbiliomycetes, P: Pezizomycotina, S: Saccharomycotina, T: Taphrinomycotina. The bootstrap support values were 100 on all branches. Pie charts based on CAFE analysis of 13,402 orthoMCL gene families indicate expanded and contracted gene families for each branch in the phylogenetic tree. The size of each pie chart is proportional to the total number of gene families that have either expanded or contracted (Figure S3). (B) Venn diagram of the predicted proteins in *M. haptotylum* and *A. oligospora* versus those of 14 other fungal species. The slight difference in number of genes between *M. haptotylum* and *A. oligospora* in each category is due to different gene copy numbers.

A phylogenomic analysis using all single copy orthologs from 16 fungal species resolved the 14 ascomycetes into the three previously identified subphyla: Taphrinomycotina (*Schizosaccharomyces pombe*), Saccharomycotina (*Candida albicans*, *Ashbya gossypii* and *Saccharomyces cerevisiae*) and Pezizomycotina [2] (Figure 2A). Taphrinomycotina is resolved as the earliest diverging clade. Also in agreement with previous studies [2], the nematode-trapping fungi of the Orbiliomycetes represent the earliest diverging clade of the Pezizomycotina, with the remaining species sampled forming a well-supported crown clade.

Taking the date of the split between ascomycetes and basidiomycetes to be 500–650 million years ago (MYA) [30], the date of the split between the Orbiliomycetes and crown clades were estimated to 400–520 MYA. The lineages of *A. oligospora* and *M. haptotylum* diverged about 14–18 MYA. Using the same calibration, the divergence between the entomopathogenic fungi *M. anisopliae* and *Metarhizium acridum* was estimated to 34–44 MYA, which is almost identical to the divergence time reported by Gao *et al.* [19] (33–43 MYA). The finding that the two nematode-trapping fungi are more closely related to each other than the two entomopathogens are to each other is unexpected, because the amino acid identity between pairs of orthologs for the nematode-trapping fungi (78.5%) was lower than for the entomopathogenic fungi (91.5%) [19]. A likely explanation for these results is that that the rate of amino acid substitutions is higher in the nematode-trapping fungi lineage than in the lineage of *M. anisopliae* and *M. acridum*. The estimation of the divergence time between *A. oligospora* and *M. haptotylum* differed substantially from the a previous study using five genes [21]. However, taxonomic assignment of one of the fossils used in the study has been questioned [22].

### Gene family expansions

Lineage-specific gene expansion has been shown to be one of the most important means of adaptation in eukaryotes [31]. To study the gene family expansions and contractions in the genomes of *M. haptotylum* and *A. oligospora*, an analysis of gene family evolution comparing 16 genomes was performed (Figure 2A). The software CAFE (Computational Analysis of gene Family Evolution) uses a maximum likelihood model to study gene family evolution while taking into account the phylogenetic relationships between the species. In total, 13,402 gene families, identified, using orthoMCL, were analyzed and the ancestral family sizes estimated with CAFE. In the branch leading to *M. haptotylum*, 848 gene families changed in size. Out of these, 326 (38.4%) of the gene families were expanded and 522 (61.6%) contracted (Figure 2A; Figure S3). Out of 806 gene families that changed in the branch leading to *A. oligospora* 362 (44.9%) were expanded and 444 (55.1%) were contracted families. The lowest proportion of expanded gene families among the filamentous ascomycetes was found in *N. crassa* branch (9%) and the highest in *M. anisopliae* (52.6%). The proportion of expanded gene families of the nematode-trapping fungi was similar to *M. oryzae* (38.0%) and higher than *M. acridum* (26.9%) (Figure 2A; Figure S3).

**Figure 3:**
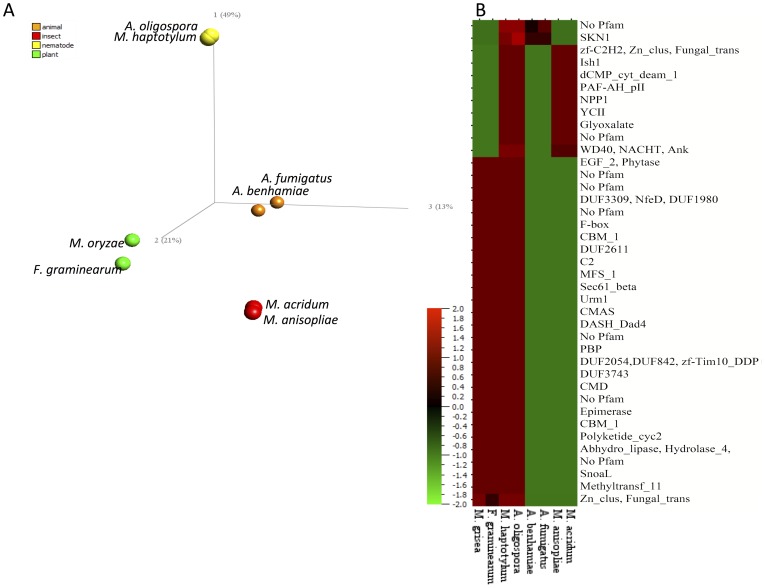
Comparison of gene families in filamentous ascomycetes. (A) Principal component analysis of eight filamentous pathogens based on gene counts from all gene families (13,402) identified by OrthoMCL clustering. Scale was log_2_(gene counts+1) and variance filtering was set to 0.2. The colors indicate the host infected by each species. Filamentous pathogens infecting plants are green (*M. oryzae*, formerly *M. grisea*, *Fusarium graminearum*), insects are red (*M. anisopliae*, *M. acridum*) and other animals (humans) are orange (*Arthroderma benhamiae*, *A. fumigatus*). (B) Heat map of expanded gene families in common between nematode-trapping fungi and other filamentous pathogens infecting plants (*M. grisea*, *F. graminearum*), insects (*M. anisopliae*, *M. acridum*) and other animals (*A. benhamiae*, *A. fumigatus*). The gene families where clustered using hierarchical clustering of log_2_(gene counts+1). Only 39 gene families passing the F-test with variance filtering of 0.2 and q-value<0.05 (false discovery rate, adjusted for multiple testing) are shown. Pfam domains present in proteins belonging to each gene family are shown to the right. Scale is log_2_(gene counts+1) .

A principal component analysis (PCA) of the expanded orthoMCL gene families in filamentous fungal pathogens grouped the species according to which host they infect (Figure 3A). The first axis (explaining 49% of the variability) separated the nematode-trapping fungi from those using other hosts including plants, insects or humans. All these species were from the crown clades of the Pezizomycotina. The second (21%) and third axis (13%) separated the species in this group into plant, insect and human pathogens. A statistical test was used to identify the gene families that contributed to the separation of the lifestyles of the fungi (Figure 3B). The nematode-trapping fungi and plant pathogens shared the largest number of expanded gene families (28 families). The nematode-trapping fungi and insect pathogens shared fewer numbers of expanded gene families and the nematode and animal pathogens the least (9 and 2 families, respectively). Only two proteins encoded by genes in the gene families (one shared with insect pathogens and one shared with plant pathogens) above matched proteins in PHI-base [29] (Table S8).

**Figure 4:**
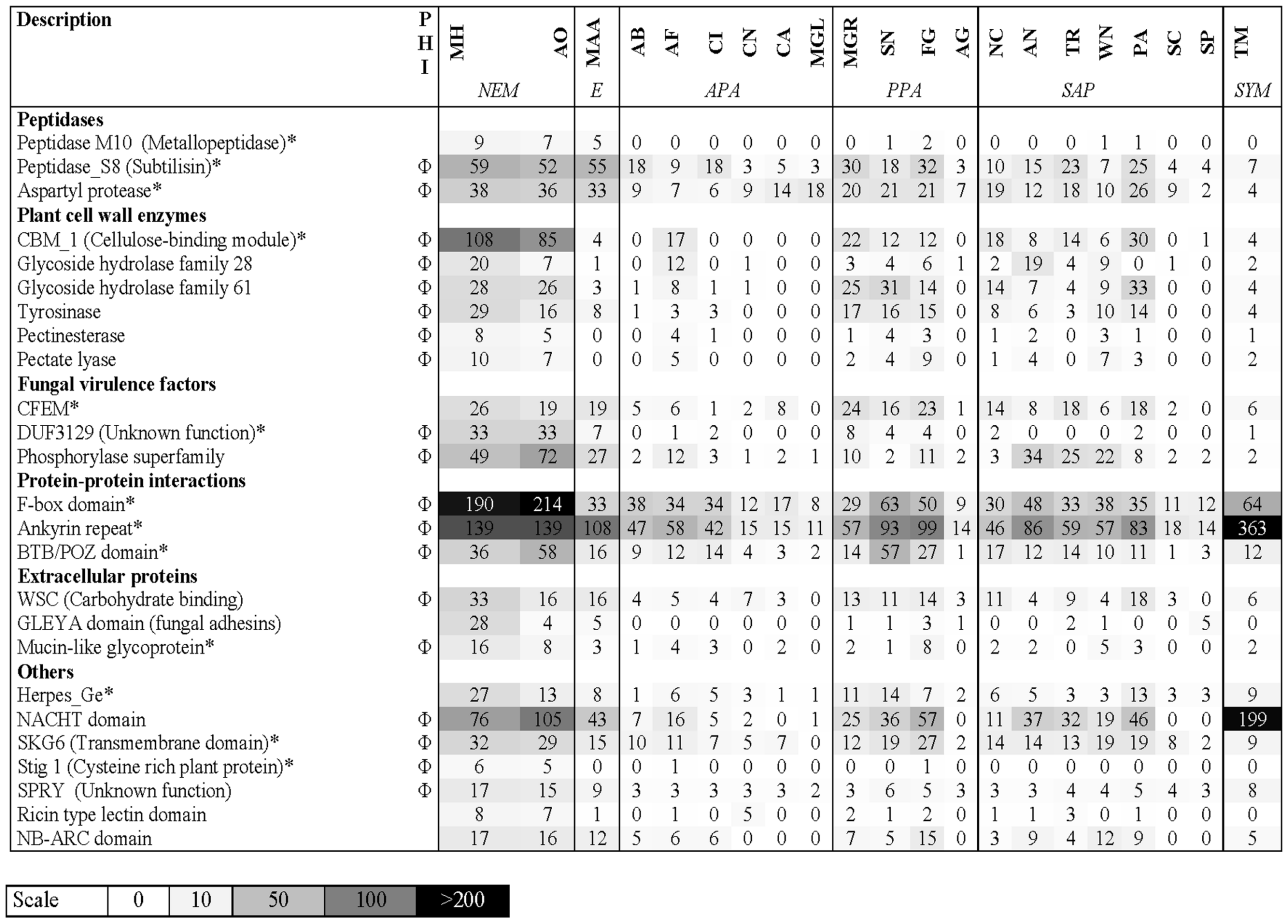
Expanded gene families in *M. haptotylum*. The number of proteins in 25 Pfam families that were found to be significantly (P<0.001) enriched in the genome of *M. haptotylum* are compared with their sizes in 20 other fungal genomes. The symbol ‘*’ indicates that the family were also found among the lineage-specific families (Figure 2B). The symbol ‘Φ’ indicates that the Pfam family contains *M. haptotylum* proteins that match proteins (BLASTP, threshold value <1E -10) in the pathogen–host interaction (PHI-base) database [29]. Species and group abbreviations: *NEM*, the nematode-trapping fungi *M. haptotylum* (MH) and *Arthrobotrys oligospora* (AO); *E*, the entomopathogenic fungus *Metarhizium anisopliae* (MAA); *APA*, animal pathogenic fungi including *Arthroderma benhamiae* (AB), *Aspergillus fumigatus* (AF), *Coccidioides immitis* (CI), *Cryptococcus neoformans* var *neoformans* (CN), *Candida albicans* (CA) and *Malassezia globosa* (MGL); *PPA*, plant pathogenic fungi including *Magnaporthe oryzae* (formerly *M. grisea*) (MGR), *Stagonospora nodorum* (SN), *Fusarium graminearum* (FG) and *Ashbya gossypii* (AG); *SAP*, the saprotrophic fungi including *Neurospora crassa* (NC), *Aspergillus niger* (AN), *Trichoderma reesei* (TR), *Emericella nidulans* (WN), *Podospora anserina* (PA), *Saccharomyces cerevisiae* (SC) and *Schizosaccharomyces pombe* (SP); *SYM*, the symbiotic fungus *Tuber melanosporum* (TM).

In addition, a separate analysis using Pfam domains was used to investigate protein domain families with functional annotation. Pfam domain families (in contrast to orthoMCL gene families) group proteins based on conserved functional protein domains. In total, 3,124 protein domain families (containing 7,455 proteins) in *M. haptotylum* and 3,782 protein domain families (containing 7,555 proteins) in *A. oligospora* were identified. 42 of the Pfam families were identified to be significantly larger or smaller in *M. haptotylum* than in other ascomycetes (Table S9). The 25 expanded protein domain families in *M. haptotylum* contained several peptidases, plant cell wall degrading enzymes and virulence factors of plant pathogenic fungi (Figure 4). Extracellular proteins and proteins involved in protein-protein interactions were also found among the significantly overrepresented gene families. Furthermore, a large fraction (19 out of 25) of the expanded Pfam families contained members that displayed significant similarities to proteins in the PHI-base (Figure 4).

A comparison of the CAFE gene families and the expanded Pfam domain families revealed strong similarities. In total, 80 out of the 326 expanded orthoMCL gene families in *M. haptotylum* contained expanded Pfam domains (22 of the 25 expanded Pfam domains were detected). In *A. oligospora*, 68 of the 362 expanded gene families contained expanded Pfam domains (19 of 25 Pfam domains). Together, the expanded gene families in the two branches of *M. haptotylum* and *A. oligospora* match all 25 expanded Pfam domains, indicating that the expanded Pfam domain families are important for the evolution of the two nematode-trapping species.

### Repeat-induced point mutations

The genomes of both *M. haptotylum* and *A. oligospora* contained a low number of repetitive elements (Table S10). RIP generates mutations in repeat regions of a genome and has been reported in several fungi [12], [15], [19], [27], including *A. oligospora* [20]. In *A. oligospora*, the RIP index was calculated using the RIP indices and RIP index scan. We performed a genome-wide RIP index analysis of *M. haptotylum* and also, for comparison, of *A. oligospora*, to test whether the RIP mechanism had contributed to the low percentage of repetitive elements. Two different RIP indices were calculated [32]. The TpA/ApT index measures the products of the RIP mutations; a higher value suggests a stronger RIP response. This index corrects for false positives in AT-biased sequences and is suitable for detecting genes that have TpA point mutations due to RIP activity. It has successfully been used to identify RIP-affected genes that are located close to TEs [33]. The RIP index, (CpA+TpG)/(ApC+GpT), which estimates the depletion of RIP targets in genes, is an alternative index commonly used [32]. The TpA/ApT index was calculated to be 1.12 in *M. haptotylum* and 1.28 in *A. oligospora* (≥0.89 indicates RIP mutations) (Figure S4); the (CpA+TpG)/(ApC+GpT) index was calculated to be 0.93 in *M. haptotylum* and 0.83 in *A. oligospora* (≤1.03 indicates RIP mutations). Both indices therefore indicate RIP activity in both genomes. A decrease in CpA dinucleotide abundance was detected in *A. oligospora* but not in *M. haptotylum*, indicating that the RIP mechanism may have different dinucleotide preferences between the two species (Figure S5).

The RIP mechanism is activated when the fungus undergoes sexual reproduction [12]. Ascomycete fungi may be heterothallic (outcrossing) or homothallic (self-crossing) [34]. Two types of compatible MAT genes (also called idiomorphs) are required for successful crossing. The *MAT1-1* idiomorph contains an alpha domain and the *MAT1-2* idiomorph contains a HMG-box domain. Only one protein with weak match to a MAT_alpha1 domain in the Pfam database (E-value 0.027) was detected in *M. haptotylum* (H072_9577) and no such match was found in *A. oligospora*. The *M. haptotylum* protein H072_9577 matched *A. oligospora* hypothetical protein (EGX48882; E-value 1E-15) using BLASTP. None of these proteins displayed significant sequence similarities to *MAT1-1* proteins in the NCBI nr database (BLASTP search). Consequently, no *MAT1-1* idiomorph was identified in the genomes of *M. haptotylum* and *A. oligospora.* The *MAT1-2* idiomorph was identified by searching for the HMG_box domain using Pfam database. In *M. haptotylum* 11 proteins were identified. Two of these (H072_9933 and H072_9576) had *MAT1-2* homologs in a BLASTP search. H072_9676 matched a hypothetical protein in *A. oligospora* (EGX48881; E-value 2E-34) and a *MAT1-2_1* protein from *Peyronellaea pinodella* (AER26933; E-value 1E-09). One homolog to H072_9933 was MAT1-2 protein variant 2 from *Cercospora beticola* (AFH56912, E-value 5E-09). In *A. oligospora* 10 proteins containing the HMG_box domain were identified and 4 of these proteins (EGX51833, EGX50317, EGX49798 and EGX49654) have homologs to *Podospora anserina* hypothetical S mat+ (*MAT1-2* idiomorph) proteins in the NCBI database. In summary, in the sequenced strains of *M. haptotylum* and *A. oligospora* only one of the idiomorphs (*MAT1-2* containing the HMG_box domain) was identified suggesting that both the *M. haptotylum* and *A. oligospora* species are heterothallic. Alternatively, the *MAT1-1* idiomorph could be located in a region of the genomes that have not been fully sequenced.

An active RIP mechanism would be expected to act on gene duplications and thereby gene families by causing rapid mutations leading to a reduction of the number of expanding families. For example, the strong RIP activity in *N. crassa* resulted in a very low percentage of expanded gene families [12]. A majority of the gene families in the *M. haptotylum* and *A. oligospora* branches are contracted (61.6% and 55.1% of families with altered sizes, respectively), indicating that an active RIP mechanism has reduced the number of expanded gene families (Figure 2A; Figure S3). However, because sequence divergence is not taken into account in the CAFE analysis, the nucleotide divergence between gene duplications was also investigated using Usearch software to identify recently duplicated genes [35]. RIP has been shown to act on duplicate sequences that are longer than 400 bp and at least 80% identical [12]. Among the 11,479 genes in *A. oligospora*, none were both longer than 400 bp and more than 80% identical. In *M. haptotylum*, we identified eight of of 10,959 gene models that were longer than 400 bp and had more than 80% identity. Five of these eight genes encode proteins with the Pfam domains related to transposons. RVP_2 (retroviral aspartyl protease) and RVT_1 (reverse transcriptase) were detected in three predicted proteins and two proteins contained the Transposase_5 domain. In conclusion, the paucity of highly similar gene duplicates strongly indicates an active RIP mechanism and shows that the expanded gene families contained genes that have diverged to the extent that they are no longer recognized by the RIP mechanism.

### Core, lineage- and species-specific genes

Comparative genomic analysis showed that the two nematode-trapping fungi contained a large number of lineage-specific and species-specific genes (Figure 2B). In total ∼62% of the genes in the genomes of *M. haptotylum* and *A. oligospora* were shared by other fungi; ∼20% of the genes were shared between the two nematode-trapping fungi and up to 16% of the genes were unique in each genome.

There was a marked difference in the size and features of genes between the core and specific groups. For example, the average size of the core genes was more than twice as large as that of the species-specific genes (the length of the encoded protein of the core, lineage-specific and species-specific genes in *M. haptotylum* were 583, 455 and 281 amino acids, respectively, and in *A. oligospora* 571, 452 and 275 amino acids, respectively). Moreover, the TEs were predominantly found among core genes rather than in the lineage-specific and species-specific genes in both species (Figure S2).

**Figure 5:**
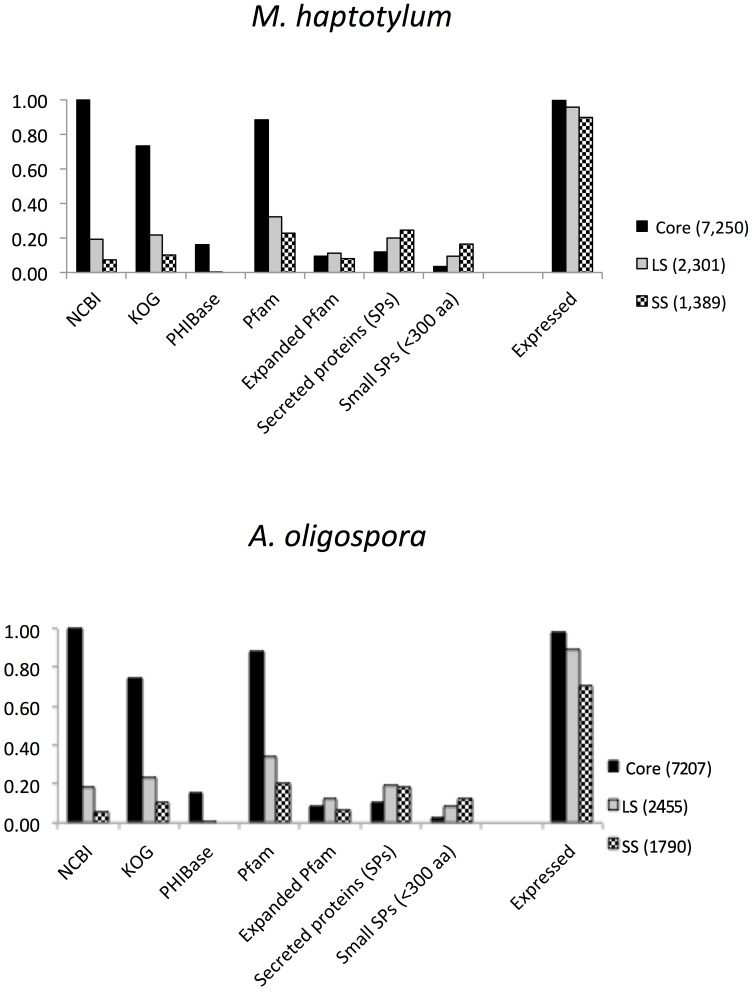
Features of core, lineage-specific (LS) and species-specific (SS) protein-coding genes in *M. haptotylum* and *A. oligospora*. The proportions of proteins among the core, LS and SS categories that have homologs in the NCBI nr database, the EuKaryotic Orthologous Groups (KOG) database and the pathogen–host interaction protein database (PHI-base) are shown. “Pfam” indicates the fraction of proteins with Pfam domains; “Expanded Pfam” indicates proteins with Pfam domains of expanded gene families (see Figure 4); “Secreted proteins (SPs)” indicates proteins with predicted signal peptide; SSPs indicates SPs with a length <300 amino acids; “Expressed” indicates the fraction of proteins supported by expression (RNASeq) data. The total number of genes in the core, LS and SS categories are shown in parentheses.

A large proportion of the lineage-specific and species-specific genes had no homologs in the NCBI nr database and many lacked Pfam domains (Figure 5). In *M. haptotylum*, 71% of the species-specific genes lacked both homologs and Pfam domains, so they can be referred to as orphans [36]. In the *A. oligospora* genome, 76% of the species-specific proteins were orphans. Moreover, the lineage-specific and species-specific groups were enriched for secreted proteins, including small (<300 amino acid) secreted proteins (SSPs) (Figure 5). Analysis also showed that many of the abundant Pfam domains among the lineage-specific and species-specific genes were found among the expanded Pfam domain families (Table S11). All of the genes in the expanded orthoMCL families shared between the nematode-trapping fungi and plant-, insect- and animal pathogens (Figure 3B) were found in the core category. Three of these genes encode SSPs and all belonged to expanded orthoMCL families shared between nematode-trapping fungi and plant pathogenic fungi from the Pezizomycotina crown group. Analysis of KOG terms of the core, lineage-specific and species-specific genes showed that the biological functions were unequally distributed among these groups. The lineage-specific and species-specific groups were enriched for genes involved in transcription, cytoskeleton, cell wall/membrane/envelope biogenesis, secretion and signal transduction. In contrast, the core regions contained homologs of genes involved in metabolism and energy production (Figure S5).

### Infection-induced gene expression in *M. haptotylum*

L1 larvae of the nematode *C. briggsae* were added to the infection structures (knobs) of *M. haptotylum* and the infection was followed by microscopy. After 4 hours of infection, approximately 98% of the added nematodes were paralyzed. This phase of the infection occurs at the time when the fungus has penetrated the nematode cuticle [37].

**Figure 6:**
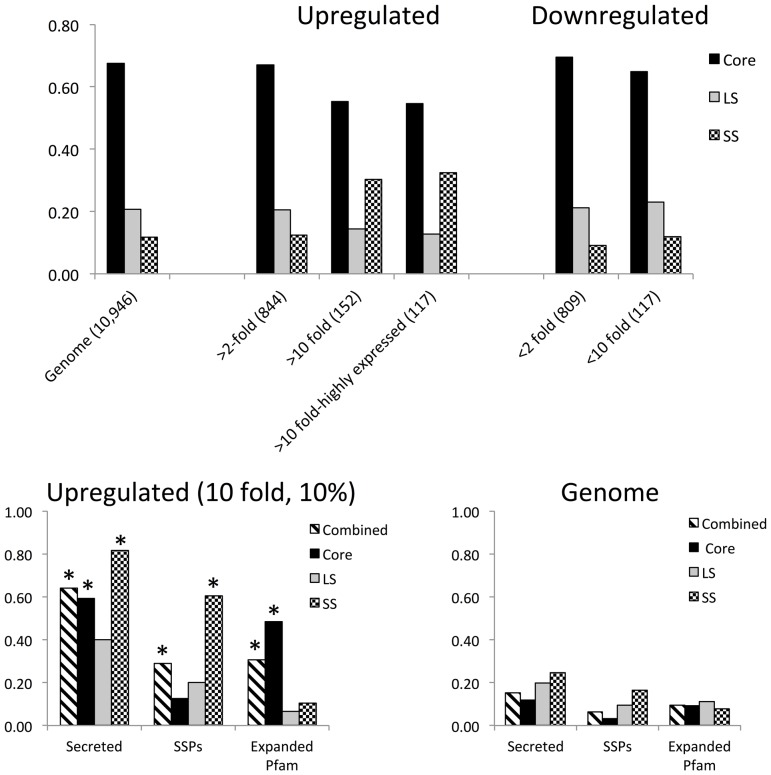
Gene expression by *M. haptotylum* during the infection of the nematode *C. briggsae*. (A) Shown is the proportion of protein-coding genes that were upregulated or down-regulated at 2-fold or 10-fold levels when comparing the expression levels in penetrating mycelium (4 hours of infection) and knobs. The genes were classified into core, lineage-specific (LS) and species-specific (SS) genes. (B) Features of the highly upregulated (>10 fold) and expressed (>10%) genes. “*” indicates the categories of genes that were significantly (P<0.001) enriched among the upregulated genes (left) as compared with their distribution in the genome (right). “Combined” includes core + LS + SS.

RNASeq analysis showed that ∼15% (1,653) of the significantly upregulated genes in *M. haptotylum* were more than two-fold up- or down-regulated during the penetration of *C. briggsae* (Figure 6A). The distribution of these genes into the core, lineage-specific and species-specific categories were similar to that of the genome. However, a significant enrichment of species-specific genes was found in the cohort of the ten-fold upregulated transcripts. Species-specific genes that are highly upregulated as well as highly expressed during infection are likely to be involved in the adaptation to parasitism in *M. haptotylum*. Therefore, we identified the most highly upregulated and most highly expressed genes in *M. haptotylum*. Of the 10% most highly expressed genes during infection (1,069 genes), 117 were more than ten-fold upregulated during the infection. In total, 38 of these 117 infection-regulated genes were specific for the *M. haptotylum* lineage, 15 were unique for the *M. haptotylum* and *A. oligospora* lineage and 64 common to other fungi (the core set). Furthermore, the cohort of the highly expressed infection-regulated genes was greatly enriched with secreted proteins (Figure 6B). Secretion signals were predicted in 75 protein sequences, i.e. 64% of the infection-related genes, which is considerably higher than the proportion of secreted proteins in the whole proteome (15%) (Figure 6B). In total 36 of the 75 secreted proteins were short (<300 amino acids) and are thus considered to be SSPs. These proteins are rich in cysteine residues, with 15 of the 36 SSPs containing at least five cysteines, indicating that the secondary structure is likely to be highly stable in the environment outside of the fungal cell.

Members of several expanded Pfam domain families were also enriched among the highly upregulated and expressed genes. In total, 36 of the 117 regulated genes (31%) were from expanded Pfam domain families, which is a significantly higher fraction than found in the whole proteome (8%). The expanded Pfam domain families were primarily encoded by the core set of genes and they contained the DUF3129, WSC, tyrosinase, mucin and peptidase S8 families (Table 2). Twenty-eight of the highly infection-regulated genes showed sequence similarities to proteins in the PHI-base, including the members of the DUF3129 Pfam domain family that encode the gas1 proteins of the rice blast fungus *Magnaporthe oryzae* [38], an extracellular cutinase (PBC1) from a plant pathogen [39], a tetraspanin homolog from *Colletotrichum lindemuthianum* [40], and *RBT4* from *C. albicans* [41]. The function of the protein encoded by *RBT4* is not known, but it contains a CAP (Cysteine-rich secretory proteins, Antigen 5 and Pathogenesis-related 1 protein) domain. None of the genes in the expanded orthoMCL families that were shared with the plant, insect or human pathogens were found among the highly upregulated and expressed transcripts during nematode infection.

**Table 2:** Number of highly upregulated or highly expressed transcripts in expanded Pfam domain families of *M. haptotylum* and *A. oligospora* during nematode infection.

	*M. haptotylum*			*A. oligospora*	
	I/K^a^	>10%^b^	M/A^c^	>10%^b^	A/M^d^
**Peptidases**					
Peptidase M10		1			
Peptidase S8	2	4	2	5	3
Aspartyl protease			1	2	1
**Plant cell wall enzymes**					
CBM_1	1	4	5		6
Glycoside hydrolase family 28		1			
Glycoside hydrolase family 61			2		2
Tyrosinase	5	10		2	1
Pectinesterase					
Pectate lyase			1		1
**Fungal virulence factors**					
CFEM		5	1	2	
DUF3129 (gas 1)	14	17	1	2	1
Phosphorylase superfamily		1	3	10	3
**Protein-protein interaction**					
F-box domain		10	3	5	4
Ankyrin repeat	1	10	5	21	5
BTB/POZ domain		2		1	4
**Extracellular proteins**					
WSC	10	12		3	
GLEYA		3	1	1	1
Mucin-like	2	3			
**Others**					
Herpes_gE	1	1		1	
NACHT domain		5	4	18	4
SKG6		3	2		1
Stig 1					
SPRY		2		3	1
Ricin type lectin domain			1	1	2
NB-ARC domain			1	1	
Total number of genes (Total in genome)	36 (117)	94 (1,069)	33 (338)	78 (1,145)	40 (335)

a “I/K” indicates the number of genes upregulated >10 fold and found among the most highly (>10%) expressed genes (Figure 6B).

b “>10%” indicates the number of genes found among the 10% most highly expressed genes on nematode infection.

c “M/A” indicates the number of genes upregulated >10 fold in the pair-wise comparison between *M. haptotylum* and *A. oligospora* (Figure 7B).

d “A/M” indicates the number of genes upregulated >10 fold in the pair-wise comparison between *A. oligospora* and *M. haptotylum* (Figure 7C).

### Comparative transcriptomics

The transcriptome of *M. haptotylum* expressed during the early stage of infection of *C. briggsae* was compared with that expressed by *A. oligospora* during infection of *C. briggsae*. The initiation of the infection process of *A. oligospora* was slightly slower and less synchronized than that of *M. haptotylum* because the traps are fewer and hence more dispersed. Thus, to probe similar stages of the infection, the 4-hour samples of *M. haptotylum* were compared with the 6-hour and 10-hour samples of *A. oligospora*.

Analysis of the sequences of the 10% most highly expressed genes in *M. haptotylum* (which included 1,069 genes) showed that these were slightly enriched with secreted proteins (not statistically significant). Among them, 200 proteins (19%) were predicted to be secreted proteins of which 84 were SSPs. The proportion of secreted proteins in the genome is 15%. Such an enrichment of secreted proteins was not observed in the transcriptome of *A. oligospora.* Seventy-two secreted proteins were identified among the 10% most highly expressed genes (in total 1,145 genes) of which 10 were SSPs. Genes from expanded Pfam domain families were highly expressed in both fungi, including the subtilisin (peptidase S8), tyrosinase, CFEM, DUF3129 (gas1), WSC and GLEYA families (Table 2).

A more detailed analysis of the regulation of genes shared between the two nematode-trapping fungi was performed by comparing the expression levels of ortholog pairs, which represent genes that have most likely evolved from a common ancestral gene. In total, 2,599 (32%) of the 8,121 ortholog pairs showed significant difference in fold change (q-value<0.01) between the two fungi (Figure 7A). Notably, the orthologs displaying the largest differences in expression values, that is, those that were more than 10-fold up- or down-regulated in the pairwise comparison between *M. haptotylum* and *A. oligospora*, were enriched with genes encoding secreted proteins, including many lineage-specific genes and SSPs (Figure 7B and 7C). Several expanded Pfam domain families were also enriched among the differentially expressed genes (Table 2). Three of the expanded orthoMCL families (Figure 3B) were also found among the differentially expressed genes. Two of these families contained Pfam domains including NPP1 (necrosis inducing protein) and Polyketide_cyc2 (polyketide cylcases/dehydrases).

**Figure 7:**
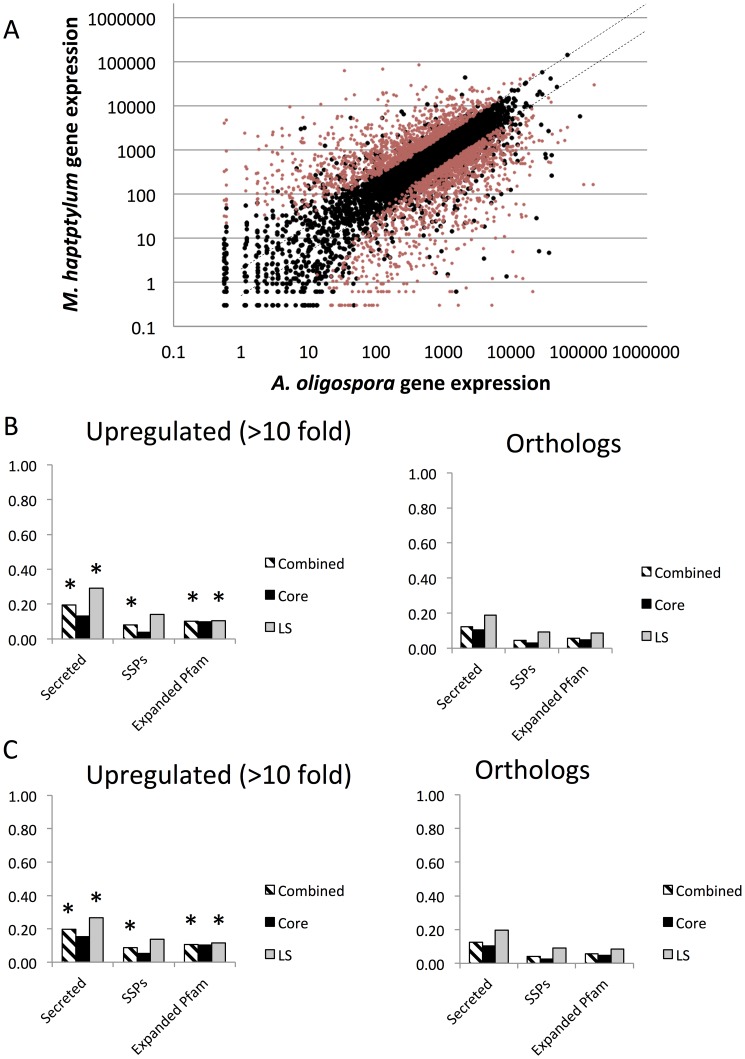
Comparison of gene expression in *M. haptotylum* and *A. oligospora* during infection of the nematode *C. briggsae*. (A) Log_10_ scatter plot of gene expression pattern between orthologs of *M. haptotylum* (mean of two replicates, 4 hours of infection) and *A. oligospora* (mean of samples from 6 and 10 hours of infection as replicates). The red circles indicate 2,599 significantly differentially expressed genes (q-value <0.01). (B) Features of *M. haptotylum* genes that are >10 fold upregulated in comparison with *A. oligospora*. (C) Features of *A. oligospora* genes that are >10 fold upregulated in comparison with *M. haptotylum*. “*” indicates the categories of genes that were significantly (P<0.001) enriched among the upregulated genes as compared with their distribution in the genome (right). “Combined” includes core + LS + SS.

### Gene clusters of SSPs

The plant pathogenic fungus *Ustilago maydis* and its close relative *Sporisorium reilianum* contain large clusters of genes encoding SSPs [42], [43]. Despite the fact that many fungal genomes have now been published, little is known about the clustering of SSPs in other fungi. To investigate whether the SSPs of *M. haptotylum* were located in such gene clusters, we used the same criteria as Kämper *et al.* [42] to identify gene clusters of secreted proteins; groups of at least three adjacent genes encoding secreted proteins or groups containing more than three genes with at most one gene encoding a non-secreted protein in between. In total, 121 such gene clusters containing 453 secreted proteins were identified in *M. haptotylum* compared with only 12 containing 79 secreted proteins in *U. maydis*. Hence, 27.2% (453/1,666) of the genes encoding secreted proteins in *M. haptotylum* were located in gene clusters, and 18.6% (79/426) of genes encoding secreted proteins in *U. maydis* were located in gene clusters. However, gene clusters are substantially larger in *U. maydis*, ranging from 3 to 26 genes, whereas the gene clusters of *M. haptotylum* consist of 3–11 genes. We further investigated how many of the secreted proteins were SSPs. Unfortunately, the total number of SSPs was not reported in the genome paper of *U. maydis* [42]. In *M. haptotylum* 27.6% of all SSPs (192 out of the 695 in the whole genome) were localized in 103 of the 121 gene clusters.

**Figure 8:**
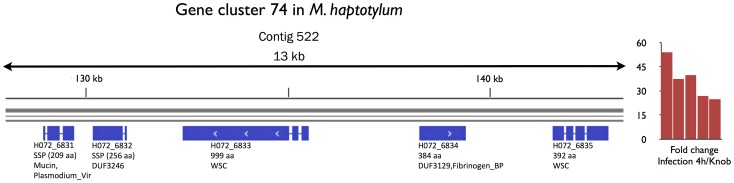
Gene cluster of secreted proteins in *M. haptotylum* that were highly expressed during nematode infection. The gene cluster (id 74) contains five of the 117 genes that were most expressed and upregulated during nematode infection (see Figure 5). All genes in the cluster contain a secretion signal; SSPs and Pfam domains are indicated below each gene. The bar chart shows the gene expression level (fold change) of each of the five genes in the cluster and is ordered in which they are found in the genome.

In *U. maydis*, 42% (33 out of 79) of the secreted genes located in gene clusters were >10 fold upregulated in tumor tissue (*in planta*) compared with axenic culture (without the host) as measured by DNA array [42]. A significantly smaller fraction (8.2%, 34 out of 453) of the secreted genes that were >10 fold upregulated in *M. haptotylum* during early infection were located in clusters. Sixteen of the 34 secreted proteins in *M. haptotylum* were SSPs, and they were located in 13 clusters. The cluster containing the largest number of upregulated SSPs was cluster 74 (Figure 8). This cluster contained two species-specific SSPs and three secreted core proteins. Four of the five genes in the cluster encoded proteins belonging to the three expanded Pfam domain families, WSC, mucin and DUF3129 (gas1).

### Sequence divergence of SSPs

**Figure 9:**
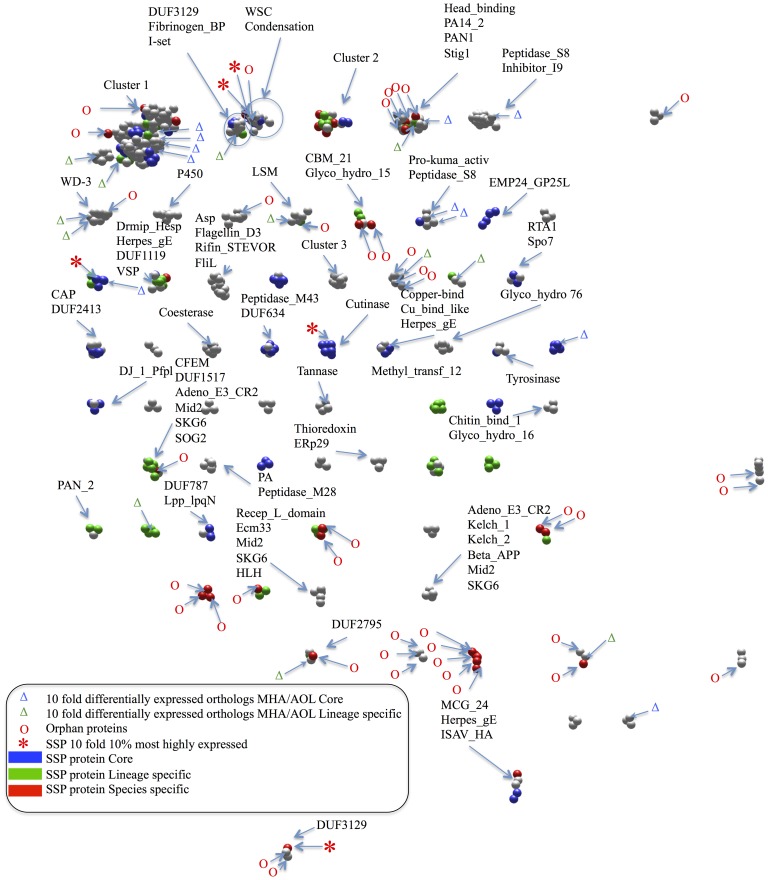
Sequence homology clustering of secreted proteins in *M. haptotylum*. Clusters of secreted proteins (nodes) containing at least three protein members are shown and Pfam domains in the clusters are indicated. The two rings indicate a cluster containing two groups of proteins with different Pfam domains and joined by only one protein. Small secreted proteins, SSPs (<300 amino acids) are colored: Blue nodes indicates proteins belonging to the core set; green are lineage-specific proteins and red nodes are species-specific proteins. Grey nodes are secreted proteins with a length of 300 amino acids or longer. Five red stars indicate SSPs that are more than 10 fold upregulated during infection versus knobs and belong to the 10% most highly expressed genes during infection. O indicates orphan sequences lacking matches to the Pfam database and other species in NCBI nr database. Blue Δ indicates core genes and green Δ indicates lineage-specific genes with 10 fold differential gene expression pattern between orthologs of *M. haptotylum* and *A. oligospora* during infection. Cluster 1 contains the following Pfam domains: Abhydrolase_2, Adeno_E1B_55K, Alpha_GJ, ArabFuran-catal, ASF1_hist_chap, BBE, CBM_1, Cellulase, Cutinase, CVNH, DUF1023, DUF1191, DUF1680, DUF291, End_N_terminal, Esterase, Esterase_phd, FAD_binding_4, Glyco_hydro_6, 7, 10, 11, 12, 16, 18, 26, 28, 30, 43, 45, 61, 62, 72, GMC_oxred_C, GMC_oxred_N, HC2, Herpes_gE, Kei1, Lipase_GDSL, LysM, Melibiase, Muc_lac_enz, Mucin, OmpW, Pec_lyase_C, Pectate_lyase, Pectinesterase, Peptidase_C8, Podoplanin, Pyr_redox, Pyr_redox_2, RE_AccI, Ricin_B_lectin, Sec10, SKN1, Stig1, Syndecan, Tyrosinase, X8 and Yip1.

The relationships between the SSPs and other secreted proteins located anywhere in the genome of *M. haptotylum* were examined using sequence homology clustering. The homologous sequences were not required to be part of any gene cluster. The genome contained 1,666 secreted proteins, including 695 SSPs. Using data from an all-against-all similarity search of the secreted proteins, 623 of the proteins were grouped into clusters containing three or more members (Figure 9). These clusters contained 181 SSPs. Out of the 181 clustered SSPs, 85 proteins were core, 60 lineage-specific and 36 species-specific proteins. Considering the total number of SSPs in these fractions, 35.2% of the SSPs in the core, 27.4% of the lineage-specific ones and only 15.3% of the species-specific ones were found in sequence homology clusters. The low numbers indicated that most SSPs do not form homology clusters, that is, they do not belong to any large group of paralogs. Notably, 23 of the 36 SSPs that were unique in *M. haptotylum* (i.e., they are found in the species-specific category) were orphans. No large clusters of orphan SSP paralogs were detected either. In fact, the largest cluster of orphan SSPs consisted of only five proteins. These proteins contained 12 conserved cysteine residues, whereas most other positions showed large sequence divergence, suggesting rapid evolution among the orphan SSPs (Figure S6). Taken together, the results from the homology cluster analysis showed that sequences encoding the SSPs are highly divergent and rapidly evolving in *M. haptotylum* and *A. oligospora*.

To investigate whether the RIP mechanism caused the rapid divergence of SSPs in *M. haptotylum*, a RIP analysis of the genes was performed separately from the RIP analysis of repeat-rich regions in the genome. The fraction of SSPs affected by RIP was calculated and compared with the fraction of all genes being affected by RIP. Analysis of the TpA/ApT index revealed that 38.2% of all *M. haptotylum* genes (4,181/10,959) have a value that is indicative of RIP activity. Using the same index, a significantly larger fraction, 76.7% of the genes encoding SSPs (533/695), were affected by a RIP mechanism. The TpA/ApT index was used to identify RIP-affected genes in the vicinity of TEs [33]. The (CpA+TpG)/(ApC+GpT) index estimating the depletion of RIP targets in the genes indicated that 31.0% of all genes, and 33.5% of the SSPs were affected by RIP. The lower percentage of RIP-affected genes is probably a result of the differential preference of target dinucleotides in *M. haptotylum* (Figure S4), where CpA is less preferred, thereby lowering the index values of the (CpA+TpG)/(ApC+GpT).

In addition to generating rapid sequence divergence, the RIP mechanism may also introduce premature stop codons by mutating CpG into TpA. Thus, such a mechanism may lead to the formation of SSPs from genes encoding longer secreted proteins. The point mutations generated by the RIP mechanism may form the stop codons TAA and TAG, but not TGA because RIP forms TA mutations and this dinucleotide is absent in the TGA stop codon. Hence, RIP mutations would be expected to give an increased frequency of TAA and/or TAG stop codons than of TGA stop codons. Among the SSPs, the ratio of genes with the TAA versus the TGA stop codons was 2.3 (358/155) and the ratio of TAG/TGA was 1.1 (177/155). The ratio of TAA/TGA and TAG/TGA in the genes (in total 6,784) with no indication of RIP activity was 1.3 (2,631/1,994) and 1.1 (2,131/1,994), respectively. Accordingly, the RIP mechanism has most likely contributed to the shortening of secreted proteins into SSPs by introducing the TAA stop codon.

## Discussion

The phylogenomic analyses presented in this study confirms previous gene-based phylogeny in placing the nematode-trapping fungi in the Orbiliomycetes as a basal branch among the filamentous growing ascomycetes (i.e. Pezizomycotina) [2], [44]. The nematode-trapping fungi divergence from the other Pezizomycotina species were estimated to 400–520 MYA, which is similar to previous estimates of 419 MYA [21]. However, despite the similar divergence time of the Orbiliaceae and the Pezizomycotina, the estimation of the divergence time in our study between *A. oligospora* and *M. haptotylum* differed substantially from that of Yang *et al.* [21]. These authors estimated that the estimated divergence between species with adhesive knobs and those with adhesive nets occurred 198–208 MYA. The divergence of the nematode-trapping fungi by Yang *et al.* relied on fewer genes (five) for the phylogenetic reconstruction, compared with the 602 genes used in our study. More importantly, the fossil records used for the dating are different between the two studies. We used the split between ascomycetes and basidiomycetes, which has been estimated at 500–650 MYA [30]; Yang *et al.* [8] used two fossil records of carnivorous fungi, dated to 100 MYA [22] and 24 MYA [45], respectively. Notably, the interpretation of the fossil record from 24 MYA has been questioned because of uncertainties in the identification of the trap structures and the assignment of the taxa [22]. In conclusion, our phylogenetic analysis using genome-wide analysis highlights the importance of reconstruction of phylogenetic trees using a large number of genes from fully sequenced genomes.

On the genomic level, there are basically three compatible mechanisms that may account for the multiple emergence and adaptations to parasitic growth in fungi [46]. First, parasitism could result from the formation of novel genes, which could have a specific role during host infection and could be acquired by gene duplication or horizontal gene transfer. Second, it could result from differences in the regulation of gene expression. Third, it could result from gene loss and deletions. The comparative genomics and transcriptomics of the closely related *M. haptotylum* and *A. oligospora* presented here provide evidence that the first two mechanisms are of major importance for the adaptation to parasitism in nematode-trapping fungi of the Orbiliomycetes. The identified increase in protein domain family sizes and large numbers of unique genes, together with the result that many of these genes were highly expressed and regulated during infection, suggest that gene duplications followed by functional diversification has been an important mechanism underlying the evolution of parasitism in *M. haptotylum* and *A. oligospora*. Moreover, the observed differential expression of orthologs in the two fungi during the early stages of infection supports the hypothesis that gene expression in nematode-trapping fungi has evolved in response to interactions with the nematode host.

The RIP mechanism has been shown to play a central role in the genome evolution of several filamentous ascomycetes belonging to the crown clade of Pezizomycotina [12], [15], [19], [27]. Signs of RIP has been reported in the *A. oligospora* genome [20]. In this study, we have examined in detail the evidence for RIP and its impact on genome evolution in nematode-trapping fungi from the Orbiliomycetes. Five observations support an active RIP mechanism in these fungi. First, the genomes of the sampled nematode-trapping fungi have a lack of repeat regions. Second, the genomes have a bias in the dinucleotide frequencies consistent with point mutations generated by RIP [32]. Third, the genomes of the nematode-trapping fungi have a low proportion of closely related gene duplicates. Our analyses showed that the genes in the expanded gene families in *M. haptotylum* and *A. oligospora* have diverged to such an extent that their sequences are no longer recognized by the RIP mechanism. Fourth, the nematode-trapping fungi have reduced number of expanded gene families in the terminal branches. The impact of an active RIP mechanism on the pattern of gene family expansion can be revealed by comparing gene family evolution in *M. anisopliae*, which lacks RIP, and *M. acridum*, which has RIP [19]. Based on these differences in RIP activity, it can be expected that *M. anisopliae* should contain more expanded gene families than contracted, as compared with *M. acridum*. Indeed, the CAFE analysis detects these differences in gene family evolution between the two *Metarhizium* species. The proportion of expanded gene families in *M. haptotylum* and *A. oligospora* branches were more similar to that of *M. oryzae* and higher than that of *M. acridum*, indicating that the RIP mechanism in the two nematode-trapping fungi is similar in strength to that observed in *M. oryzae* [27], but not as strong as detected in *N. crassa* [12]. Fifth, the strength of the RIP mechanism may be partly related to how often a particular species undergoes a sexual cycle [15]. Sexual stages have been detected only in a few species of nematode-trapping fungi like *A. oligospora* [47]. For the first time, we report on MAT genes in nematode-trapping fungi. The fact that the genomes of *A. oligospora* and *M. haptotylum* only contained one of the MAT idiomorphs strongly suggests that both species are heterothallic. The observation of an active RIP mechanism in the Orbiliomycetes, suggest that RIP evolved early in the evolution of filamentous ascomycetes, and that this mechanism has subsequently been lost in certain lineages such as those leading to *M. anisopliae*.

The most striking example of gene duplications and rapid diversification of infection-expressed genes in the genomes of *M. haptotylum* and *A. oligospora* were found among the SSPs. The first SSP that was functionally characterized in detail was Pep1 in *Ustilago maydis* [48]. Pep1 is a secreted effector protein that suppresses the plant defense responses. The importance of SSPs for successful infection of the host has been reported for many fungi belonging to the Pezizomycotina crown group, including the insect pathogen *Beauveria bassiana* [49] and the plant pathogens *Blumeria graminis* f.sp. *hordei* [50] as well as fungi from other taxonomical groups, such as *Melampsora larici-populina* (Basidiomycota) and *Puccinia graminis* (Basidiomycota) [51]. Despite the rapid diversification of SSPs, expanded gene families that were shared between nematode-trapping fungi and pathogens from the Pezizomycotina crown group contained genes encoding SSPs. All these genes encoding SSPs belonged to families that were shared with plant pathogens. Interestingly, it appears that expansion of SSPs related to pathogenicity is an evolutionary strategy used primarily by insect and plant pathogens, whereas the genomes of animal pathogens (with the exception of entomopathogens) are not enriched for genes encoding SSPs [52].

Fungal gene clusters of genes encoding secreted proteins have been reported for *U. maydis* and its close relative *Sporisorium reilianum* [42], [43] but have not been demonstrated in other pathogenic fungal genomes [15], [53]–[55]. A large number of gene clusters was revealed in *M. haptotylum*. However, the size of the clusters (number of genes) and the proportion of genes encoding SSPs was lower than in *U. maydis* [42]. A large percentage of the SSP-encoding genes in *U. maydis* were co-regulated and highly expressed during infection. In contrast, most of the highly upregulated genes encoding SSPs in *M. haptotylum* were not located in the gene clusters. Hence, while the gene clustering appear to be important for secreted proteins in general, the organization of SSPs into gene clusters may be less important for their function during infection in *M. haptotylum*. One noteworthy exception was, however, gene cluster 74. The genes in this gene cluster were all highly upregulated during infection and included virulence genes associated with adhesion (mucin and WSC) as well as DUF3129 (gas1), and hence the cluster provides an excellent candidate for future deletion experiment in *M. haptotylum* similar to the gene cluster deletions performed in *U. maydis* [42].

Two major mechanisms have been proposed for the origin of SSPs in fungal genomes [42], [56]. Firstly, TEs may increase the frequency of genome rearrangements and thereby increase the rate of gene duplication. Transposons were shown to be very frequent in the genomes of *Leptosphaeria maculans* [57], *Mycosphaerella fijiensis* and *Cladosporium fulvum* [33], leading to a dramatically larger genome size. Enrichment of SSPs in repeat-rich regions was detected in *L. maculans* but not in *M. fijiensis* or *C. fulvum*, indicating that the prolific TEs may under certain conditions lead to increased numbers of SSPs. However, the paucity of transposons and the low abundance of repetitive elements suggest that TEs did not play a major role in the evolution of SSPs in the nematode-trapping fungi. The second mechanism leading to gene duplications is unequal crossover during meiosis [58], [59]. We detected frequent tandem duplications of genes in the genome of *M. haptotylum* and a proportion of these genes were SSPs. Accordingly, we propose that unequal crossover is a major mechanism for the origin of SSP coding genes in *M. haptotylum*. In the genome of *L. maculans*, RIP activity has been shown to cause rapid divergence of SSP effectors located in close vicinity to TE-rich regions [57]. Despite the fact that such regions were not found in *M. haptotylum*, the genes encoding SSPs showed evidence of RIP activity, as revealed by a biased TpA/ApT index. The majority of the SSP sequences that were highly expressed and highly upregulated during infection have this RIP signature. In addition, we have shown that RIP can generate premature stop codons and thereby generate novel SSP coding genes from longer secreted proteins.

Taking these results together, we propose the following model for the origin and diversification of SSPs in nematode-trapping fungi. Gene duplications through unequal crossover generated a large number of SSPs. Following such duplications, the SSPs underwent rapid diversification through the RIP mechanism. The lack of SSP paralogs in *M. haptotylum* can be explained by a relatively slow rate of gene duplication in combination with a rapid divergence of the duplicated genes through RIP. In contrast, fungi such as *M. larici-populina* with large families of SSP paralogs have a fast rate of gene duplications mediated through transposons and no detected RIP activity [51]. The proposed mechanism of origin and diversification of SSPs is general and may apply to any species with an active RIP mechanism. Evolution of SSPs by gene duplication without the involvement of TEs in combination with their diversification by RIP has previously not been described in pathogenic fungi.

Several of the expanded Pfam domain families that were highly expressed during nematode infection have been identified in a previous study of *M. haptotylum* [26]. They include the subtilisins, the CFEM and the DUF3129 family (gas1). Many of the families containing upregulated genes contain genes that have been shown to be involved in the pathogenicity of animal and plant pathogenic fungi [29]. Aspartyl proteases are expressed by many pathogenic ascomycetes during infection. For example, *C. albicans* has at least 10 aspartyl proteases, which contribute to the infection by degrading the host cell surface, facilitating adhesion and degrading the host tissues for nutrition [60]. Tyrosinases are involved in the synthesis of melanin pigments [61]. The production of melanin has been shown to be essential in microbial pathogenesis, as it provides protection against host defense mechanisms and contributes to virulence in many animal and plant pathogenic fungi [62]–[65]. Tyrosinases can also oxidize protein- and peptide-bound tyrosyl residues, resulting in the formation of inter- and intramolecular crosslinks between peptides, proteins and carbohydrates [66]. It has been speculated that such enzymes could be involved in the adhesion mechanism of nematode-trapping fungi by cross-linking and stabilizing the proteins and polymers present in the extracellular layer mediating the binding between the trap and cuticle [10]. Other putative components of the extracellular adhesins might be found among the WSC and GLEYA Pfam domain families. Several genes encode proteins with features of known fungal adhesins, including signal peptide tandem repeats and predicted *O*-glycosylation sites [67] [68].

Despite the fact that there was a strong phylogenetic signal in the expansion of the orthoMCL gene families, our analysis showed that several of the expanded families in nematode-trapping fungi found in the basal clade of Pezizomycotina were also expanded in the plant, insect and animal parasitic fungi positioned in the crown clades of the subphylum. Some of these shared genes were slightly regulated in the knobs and mycelium of *M. haptotylum* and one encoded an SSP (Table S8). However, none of them were found among the highly upregulated and highly expressed transcripts during the early stages of nematode infection, which suggest that they are not of major importance for the interaction with the nematode hosts. It has been observed that nematode-trapping fungi display an extensive plasticity in the morphology and function of infection structures. For example, within one single species, namely *A. oligospora*, not only adhesive nets are formed but also appressoria in the rhizosphere of agricultural crops and hyphal coils around hyphae of other fungi [69]–[71]. It remains to be determined whether the genes in families shared between the nematode-trapping fungi and the other parasitic fungi are expressed and regulated in the interaction and colonization of non-nematode hosts, including plants.

In conclusion, we have used comparative genomics and transcriptomics to decipher the genomic mechanisms leading to the evolution of parasitism in nematode-trapping fungi. We have identified two genomic mechanisms that are likely to have been of major importance during the evolution of parasitism in nematode-trapping fungi: the formation of novel genes through gene duplication and the differential regulation of existing genes identified by differential expression of orthologous genes. In *M. haptotylum*, we have identified enrichment of SSPs that were highly and differentially expressed during the infection. We propose an evolutionary mechanism for the duplication and divergence of these SSPs. The high expression of SSPs and other secreted proteins revealed a remarkable similarity in the infection mechanisms of nematode-trapping fungi, and plant and insect pathogenic fungi. In addition, the gene family analysis revealed that nematode-trapping fungi shared more expanded protein families with the plant pathogenic fungi compared to other pathogenic fungi such as insect and other animal pathogens.

## Materials and Methods

### Fungal strain and DNA preparation

*Monacrosporium haptotylum* (strain CBS 200.50) was maintained on corn meal agar 1∶10. Mycelium was grown in liquid medium (soya peptone 0.5% w/v) and incubated at room temperature on a shaker at 200 rpm for 7 days. The mycelium was harvested by filtering and then ground in liquid nitrogen. Genomic DNA was extracted using the Qiagen Plant Maxi Kit according to the manufacturer's instructions. DNA was precipitated with ethanol and dissolved in TE buffer.

### Genome sequencing and assembly

The whole genome of *M. haptotylum* was sequenced with 454 pyrosequencing technology using a titanium shotgun protocol (XLR70) at KTH Stockholm and paired end sequencing of 3 kb insert libraries at the DNA Sequencing Facility at Lund University. The reads were assembled using the Newbler gsAssembler 2.3 software program (Roche/454 Life Sciences).

### Gene prediction and annotations

Gene models were predicted with the *ab initio* predictor GeneMark-ES [72]. Gene models that translated to peptides shorter than 48 amino acids were removed. The program Pfamscan was downloaded from the Sanger Centre FTP site (ftp://ftp.sanger.ac.uk/pub/databases/Pfam/Tools/) together with Pfam databases and hidden Markov model (HMM) libraries [73]. The Pfamscan tool was locally installed, and the predicted gene models were scanned using the Pfamscan.pl with an E-value threshold of 0.05. Predicted protein sequences were also mapped to the KOG classification system [74], [75]. Transfer RNAs were predicted by the Aragorn [76] and tRNASCAN-SE [77] programs using default settings. For comparison of the tRNAs with the *M. haptotylum* we also predicted the tRNA from the *A. oligospora* genome. Prediction of signal peptide sequences were performed using the SignalP 3.0 [78]. The genomes of *A. oligospora*, *M. anisopliae*, *A. fumigatus* and *N. crassa* were reanalyzed to identify signal peptides. Virulence-related genes were identified by BLASTP [79] similarity searches against the PHI-base database version 3.2 [29] using a cutoff of <1E-10. Core, lineage-specific and species-specific proteins were identified by BLASTP (cutoff <1E-10) similarity search against *M. haptotylum*, *A. oligospora* and other genomes (Figure 2B) using low complexity filtering. Low complexity proteins were not classified into core lineage- or species-specific proteins. Orphans were sequences that have no matches in the Pfam database and no homologs to sequences in other organisms as revealed by BLASTP searches against the NCBI nr database (cutoff <1E-10). Detection of MAT genes in *M. haptotylum* was performed by identifying proteins containing the Pfam domains MAT_alpha1 and HMG-box. Homologs were identified using BLASTP against NCBI nr database and the results were manually investigated. Percentage nucleotide identity was calculated using Usearch 6.037 [35] with cluster_fast option and an identity of 80%.

### Ortholog identification and phylogenomic analysis

Results from BLASTP (cutoff <1E-10) were used as input for clustering the sequences with orthoMCL version 2.02 [28]. The orthoMCL clusters were used for identifying gene families. Tandem duplicated genes in these families were manually identified. A custom Perl script was used to perform 1,000 permutations of the gene order to generate random sets with the same gene family distribution. For the phylogenomic analysis, only families containing exactly one gene copy for each of the 16 genomes were used, because families with paralogs may hinder correct phylogenetic inference. In total, 602 families containing 9,632 orthologs were identified and the proteins aligned using ClustalW [80]. The sequences were concatenated and poorly aligned regions of each alignment were removed using Gblocks [81]. The trimmed alignment was subsequently used for phylogenetic reconstruction using maximum likelihood method with the Dayhoff amino acid substitution model implemented in PhyML 3.0 [82] with 1,000 bootstrap replicates at the BioPortal computer cluster, Oslo University. BEAST 1.7.0 [83] was used to estimate the divergence time between *A. oligospora* and *M. haptotylum* using a Yule tree prior and lognormal relaxed clock rates. Divergence times was calculated by using the estimated splitting time between the Ascomycota and Basidiomycota branches (500 and 650 MYA) as a calibration point [30]. An ultrametric tree based on the PhyML tree was generated using Mesquite 2.75 [84]. Gene family expansions and contractions were estimated with the CAFE (Computational Analysis of gene Family Evolution) software version 3.0 [85] using the ultrametric tree and the orthoMCL gene families as input. The tree and expansion/contraction data were displayed using the iTOL web tool [86]. To rescale the data prior PCA and cluster analysis, orthoMCL families were log_2_(gene counts+1) transformed. The PCA plot with variance filtering 0.2 and heat maps using group categories (F-test) with variance filtering <0.2 and q-value <0.05 (false discovery rate, adjusted for multiple testing) were generated using the Qlucore Explorer version 2.2 (Qlucore AB, Lund, Sweden).

### Repetitive elements and transposons

Repetitive elements in the genome assembly were analyzed using Repeat Masker (version 3.2.8) using cross match an implementation of the Smith Waterman-Gotoh algorithm [87], Tandem Repeats Finder [88] and Repeat Scout [89]. RIP index was determined with the software RIPCAL by reference against the non-repetitive control families [32]. Stop codons for all genes were calculated. Putative TEs were identified by the Transposon-PSI [90] a program that performs tBLASTn searches using a set of position-specific scoring matrices (PSSMs) specific for different TE families. The genome positions of the transposons were evaluated for identification of clusters of transposons computationally as well as visually using IGV viewer version 2.2 [91]. Clusters were defined as at least five transposons located on the same contig in the genome.

### Transcriptome analyses

To validate the prediction of gene models in *M. haptotylum*, the transcriptome expressed by the fungus when grown on liquid medium was sequenced using the 454 technology. The mycelium was grown in aerated liquid medium as previously described [24]. The mycelium was quickly collected by filtering and directly dropped into a clean mortar filled with liquid N_2_ and homogenized using a pestle. The resulting powder was collected into 50 ml Falcon tubes and stored at −80°C until use. Total RNA was isolated using the RNeasy Plant Mini Kit (Qiagen) according to the manufacturer's instructions, and using the RLC buffer. Total RNA was eluted in either 60 or 100 µl of H_2_O and stored at −20°C until use. For quality and concentration assessments all samples were analyzed using a 2100 Bioanalyzer and the RNA 6000 Nano kits (Agilent). After total RNA purification, mRNA was isolated from each sample using approximately 100 µg and the absolutely mRNA Purification Kit (Agilent). The purified mRNA was then used as starting material in the cDNA Library Preparation protocol (GS FLX Titanium Series) provided by 454/Roche. In total the sequencing generated 422,883 reads that were mapped to the genome using gsMapper 2.6 (Roche). In order to compare the transcriptome with the gene models and to avoid mismatches due to reads located in the untranslated regions (not predicted by GeneMark), the reads from the pyrosequencing were assembled using gsAssembler 2.6 and subsequently mapped to the predicted gene models using BLASTN [79]. In addition, previously generated EST sequences [25], [26] were mapped to the genes as well as the genome using BLASTN.

The transcriptome expressed during infection of nematodes by *M. haptotylum* and *A. oligospora* was examined using the nematode *C. briggsae* (strain A16) as a host. Eggs were obtained by treating *C. briggsae* with 1% NaOCl in 580 mM NaOH for 5 minutes and washed with water. Hatched L1 larvae were collected the next day. Knobs of *M. haptotylum* were isolated and were incubated at room temperature with the L1 larvae on water agar plates as previously described [26]. The infection was followed under a light microscope and infected nematodes were collected after 4 h of incubation. Duplicate samples of knobs and knobs infecting *C. briggsae* were analyzed. Infection experiments with *A. oligospora* (strain ATCC 24927) were performed by growing the fungus on dialysis membranes placed on the surface of low nutrient mineral salt (LNM) medium. Traps (nets) were induced by adding *Panagrellus redivivus* nematodes [92]. After several days when the nematodes were completely digested and the traps formed, approximately 75–100 L1 larvae of *C. briggsae* were added to the dialysis membranes. The infection was followed in a light microscope and fungal mycelia, traps and infected nematodes were collected after 6 and 10 h of infection. Total RNA was extracted and sent for sequencing at GATC Biotech AG, Konstanz, Germany. After reverse transcription into double-stranded cDNA for tag preparation according to the massively parallel signature sequencing protocol [93] it was sequenced using HiSeq2000 (Illumina Inc.) in single read mode with a length of 50 bp. More than 87 million reads were sequenced for the *A. oligospora* samples and 82 million reads for the *M. haptotylum* samples. The reads were mapped to the corresponding *M. haptotylum* and *A. oligospora* genome as well as to *C. briggsae* using the Burrows Wheeler Aligner (BWA) software with default settings [94]. The transcript abundances were normalized and significantly differentially expressed genes (q-value) were identified using the R package DESeq [95]. Two replicates were used for each fungus; for *M. haptotylum* two samples after 4 hours of infection and for *A. oligospora* after 6 and 10 hours of infection (1 sample each). Although replicates for each time point in *A. oligospora* would be preferred, using two different time points as replicates, as done in this study, increase the stringency of the analysis since any variation between *A. oligospora* time points will increase the variance within the sample and thereby decrease the number of significantly differentialy expressed orthologs between the two species. This approach is similar to the approach suggested by in the DESeq manual. Bidirectional best BLAST hits (BLASTP cutoff 1E-10) were used to identify pairs of proteins (orthologs) for comparative transcriptome analyses.

### Gene clustering and homology-based clustering of secreted proteins

Gene clusters were identified in the genome of *M. haptotylum* using a procedure described by Kämper *et al*. [42]. A homology-based clustering of secreted proteins was performed by using an all-against-all BLASTP [79] similarity search (cutoff <1E-10). The network visualization program Biolayout Express 3D version 2.2 [96] was used to analyze the sequence homology clusters containing at least three secreted proteins.

### Accession numbers

The whole genome sequencing and transcriptome data of *M. haptotylum* have been deposited at DDBJ/EMBL/GenBank under Bioproject PRJNA186729 and the *A. oligospora* transcriptome data under BioProject PRJNA196395. This Whole Genome Shotgun project has been deposited at DDBJ/EMBL/GenBank under the accession AQGS00000000. The version described in this paper is the first version, AQGS01000000.

## Supporting Information

Dataset S1 Amino acid sequence alignment of 602 orthologs from 16 fungal genomes in Nexus format. The alignments from 9,632 orthologs were trimmed using Gblocks [81] giving a total of 199,402 amino acids for each of the 16 genomes. The alignment was used to generate the phylogenetic trees in Figure 2A and Figure S3.
(TXT)

Figure S1 Functional classification and comparison of *M. haptotylum* (MH) and *A. oligospora* (AO) proteins by KOG categories. In total, 71% (7,783) of the gene models in *M. haptotylum* and 69% (7,897) in *A. oligospora* had matches with the KOG database. Each circle represents the fraction of genes represented in each of the categories for each genome. The gene numbers are also shown. (Gene models with multiple classes were excluded from the analysis.)
(TIFF)

Figure S2 Transposable elements in *M. haptotylum* and *A. oligospora*. The number of transposable elements (TEs) identified in the predicted gene models of *M. haptotylum* and *A. oligospora* is shown. The graph at the bottom shows the number of TEs in core, lineage-specific (LS) and species-specific (SS) genes. The total number of TEs identified in the genomes of *M. haptotylum* and *A. oligospora* are shown in Table S5.
(TIFF)

Figure S3 Expanded and contracted gene families. Rooted maximum likelihood tree constructed from 602 single copy orthologous proteins using the Dayhoff amino acid substitution model (Figure 2A). Each branch is labeled with number of expanded/contracted gene families (Total number of gene families was 13,402). The bootstrap support was 100 for all branches in the tree.
(TIFF)

Figure S4 Fold changes in dinucleotide abundance for all repeat families in *M. haptotylum* and *A. oligospora* compared with non-repetitive control sequences on a log10 scale. This confirms the expected pattern of CpA→TpA type RIP mutations: high TpA and low TpG abundance. The difference in CpA differs between the two species.
(TIFF)

Figure S5 Functional classifications of core, lineage- and species-specific protein-coding genes in *M. haptotylum*. The proportions of proteins that have homologs in the the EuKaryotic Orthologous Groups (KOG) of proteins are shown. The total number of KOG proteins in the core, lineage-specific (LS) and species-specific (SS) categories are shown in parentheses. Protein sequences with multiple hits were excluded from the analysis.
(TIFF)

Figure S6 Amino acid representation of a cluster of five small secreted orphan proteins. The largest cluster containing only orphan SSPs was chosen (Figure 7). In the alignment, 12 cysteine residues were highly conserved while the rest of the positions were more variable. The signal peptide cleavage site was predicted between positions 20 and 21.
(TIFF)

Table S1 Genome statistics of *M. haptotylum*.
(DOCX)

Table S2 Mapping of transcriptome sequences against the genome assembly and gene models of *M. haptotylum*.
(DOCX)

Table S3 Fungal genomes analyzed in this study.
(DOCX)

Table S4 tRNA genes along with their respective anticodons present in *M. haptotylum*.
(DOCX)

Table S5 Families of transposable elements identified in the genomes of *M. haptotylum* and *A. oligospora*.
(DOCX)

Table S6 Virulence-associated proteins in the genomes of *M. haptotylum* and *A. oligospora*.
(DOCX)

Table S7 Major protein families of *M. haptotylum* implicated in pathogen–host interactions.
(DOCX)

Table S8 Expression of expanded orthoMCL gene families in *M. haptotylum* that were also expanded in plant pathogenic, insect pathogenic and animal (i.e. human) pathogenic fungi.
(DOCX)

Table S9 Gene families significantly expanded and contracted in nematode-trapping fungi in comparison with 19 other fungal species.
(DOCX)

Table S10 Repetitive sequences identified in the genomes of *M. haptotylum* and *A. oligospora*.
(DOCX)

Table S11 The most abundant Pfam domains identified among the lineage- and species-specific proteins of the nematode-trapping fungi *M. haptotylum* and *A. oligospora*.
(DOCX)
