# Supplementary figures and images for: Genomic Mechanisms Accounting for the Adaptation to Parasitism in Nematode-Trapping Fungi
Source: PLoS Genet. 2013 Nov 14;9(11):e1003909. doi: 10.1371/journal.pgen.1003909 (PMC3828140; doi:10.1371/journal.pgen.1003909)

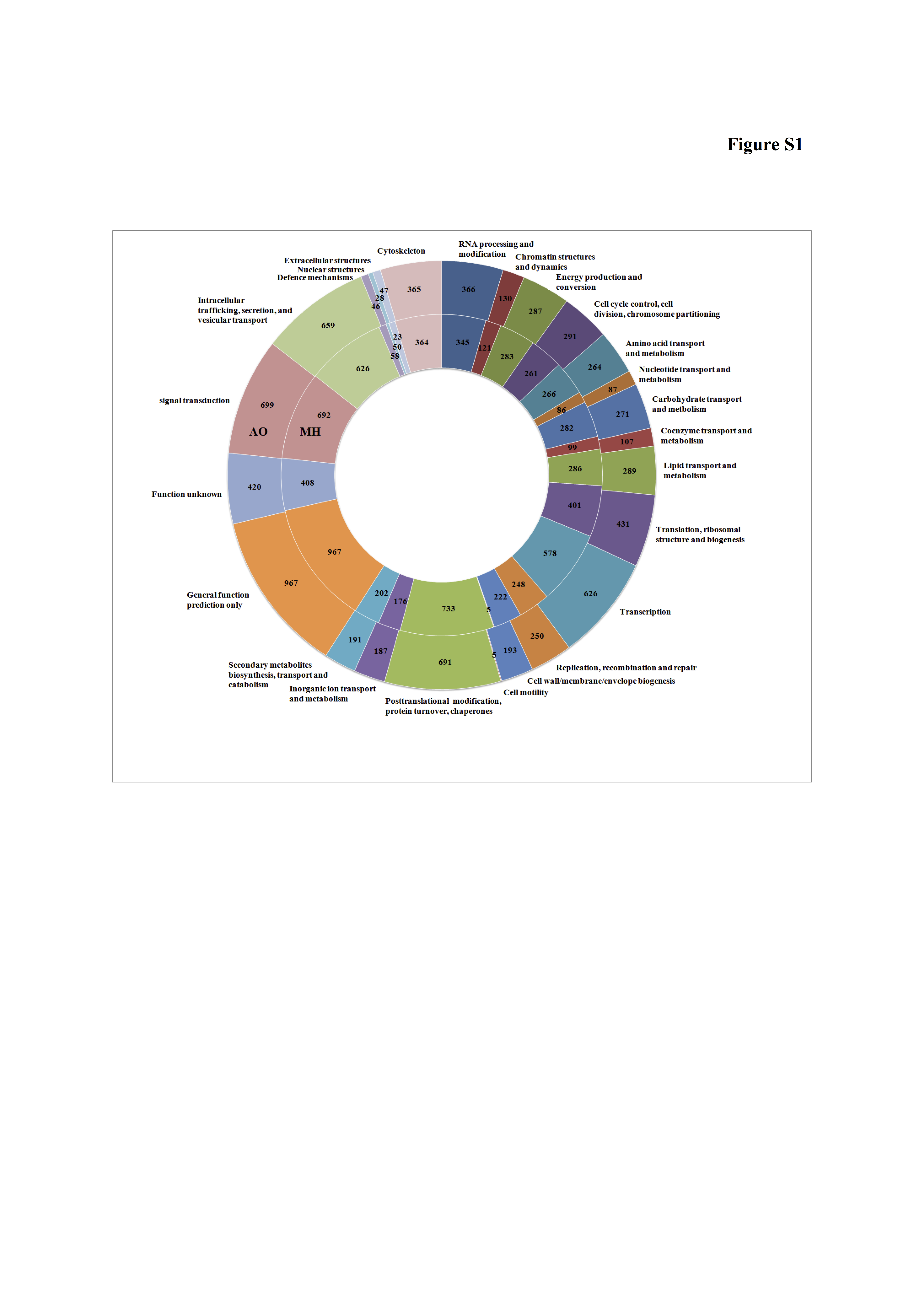

Supplement: Figure S1 — Functional classification and comparison of M. haptotylum (MH) and A. oligospora (AO) proteins by KOG categories. In total, 71% (7,783) of the gene models in M. haptotylum and 69% (7,897) in A. oligospora had matches with the KOG database. Each circle represents the fraction of genes represented in each of the categories for each genome. The gene numbers are also shown. (Gene models with multiple classes were excluded from the analysis.) (TIFF) [file pgen.1003909.s002.tif]

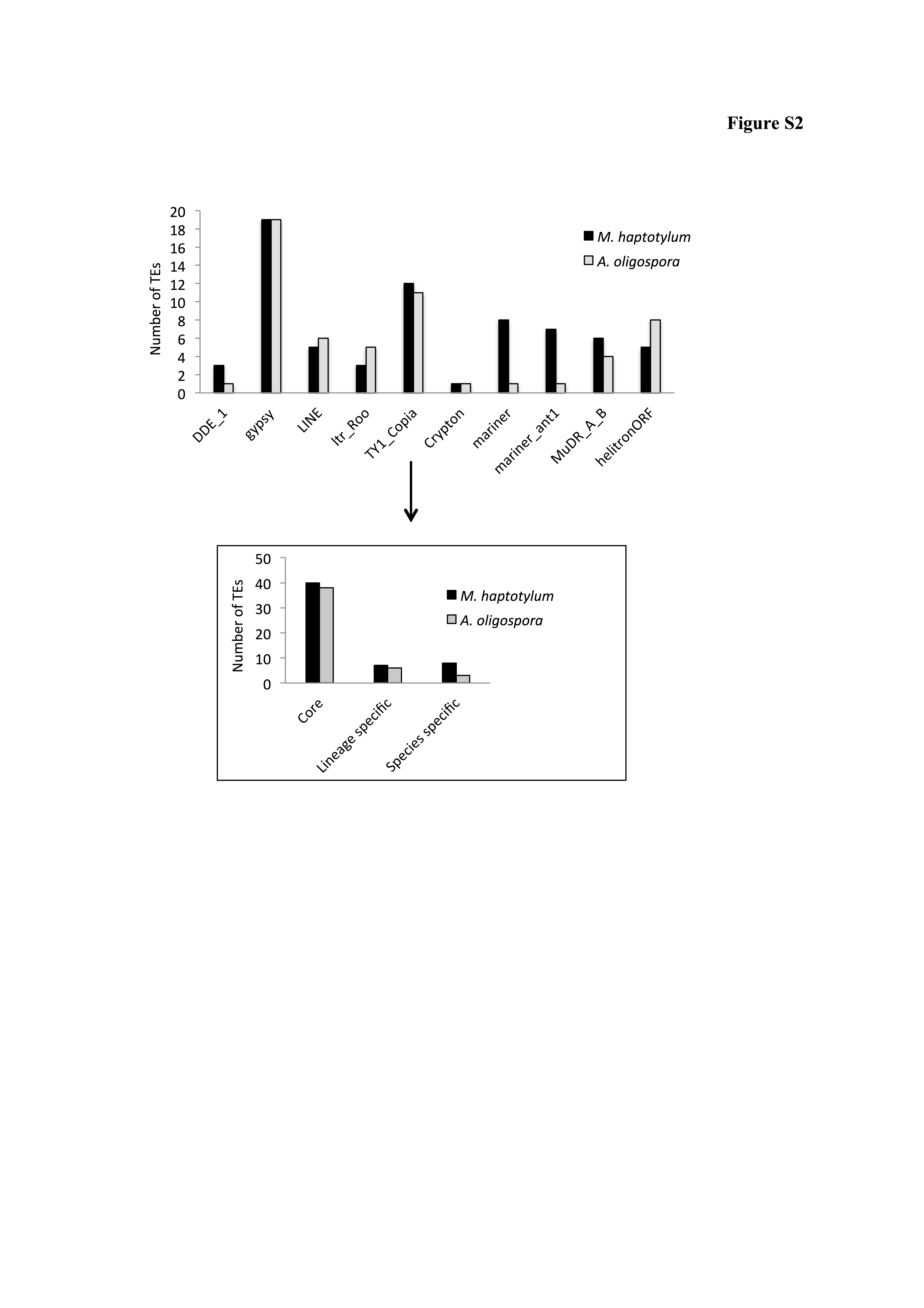

Supplement: Figure S2 — Transposable elements in M. haptotylum and A. oligospora. The number of transposable elements (TEs) identified in the predicted gene models of M. haptotylum and A. oligospora is shown. The graph at the bottom shows the number of TEs in core, lineage-specific (LS) and species-specific (SS) genes. The total number of TEs identified in the genomes of M. haptotylum and A. oligospora are shown in Table S5. (TIFF) [file pgen.1003909.s003.tif]

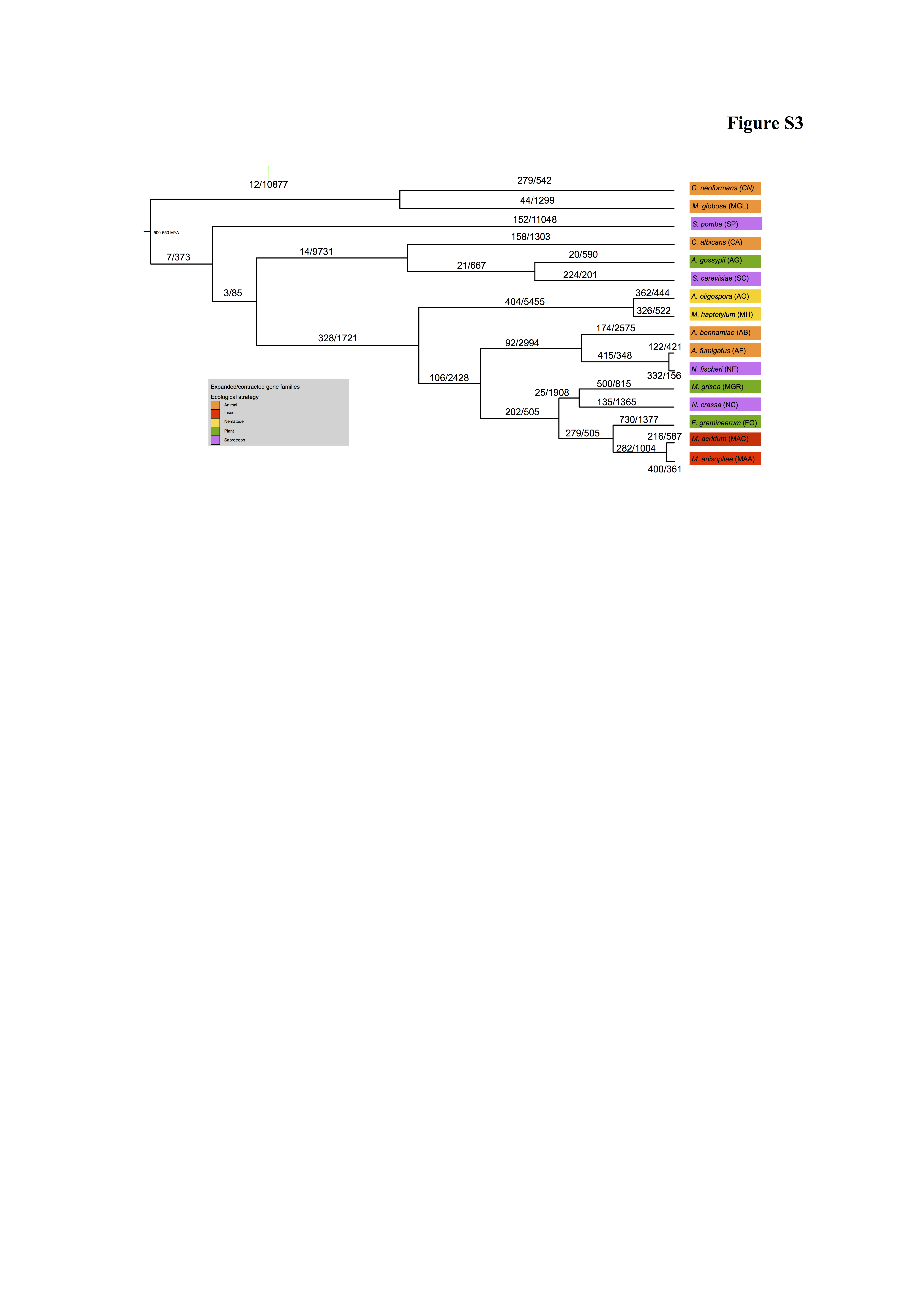

Supplement: Figure S3 — Expanded and contracted gene families. Rooted maximum likelihood tree constructed from 602 single copy orthologous proteins using the Dayhoff amino acid substitution model (Figure 2A). Each branch is labeled with number of expanded/contracted gene families (Total number of gene families was 13,402). The bootstrap support was 100 for all branches in the tree. (TIFF) [file pgen.1003909.s004.tif]

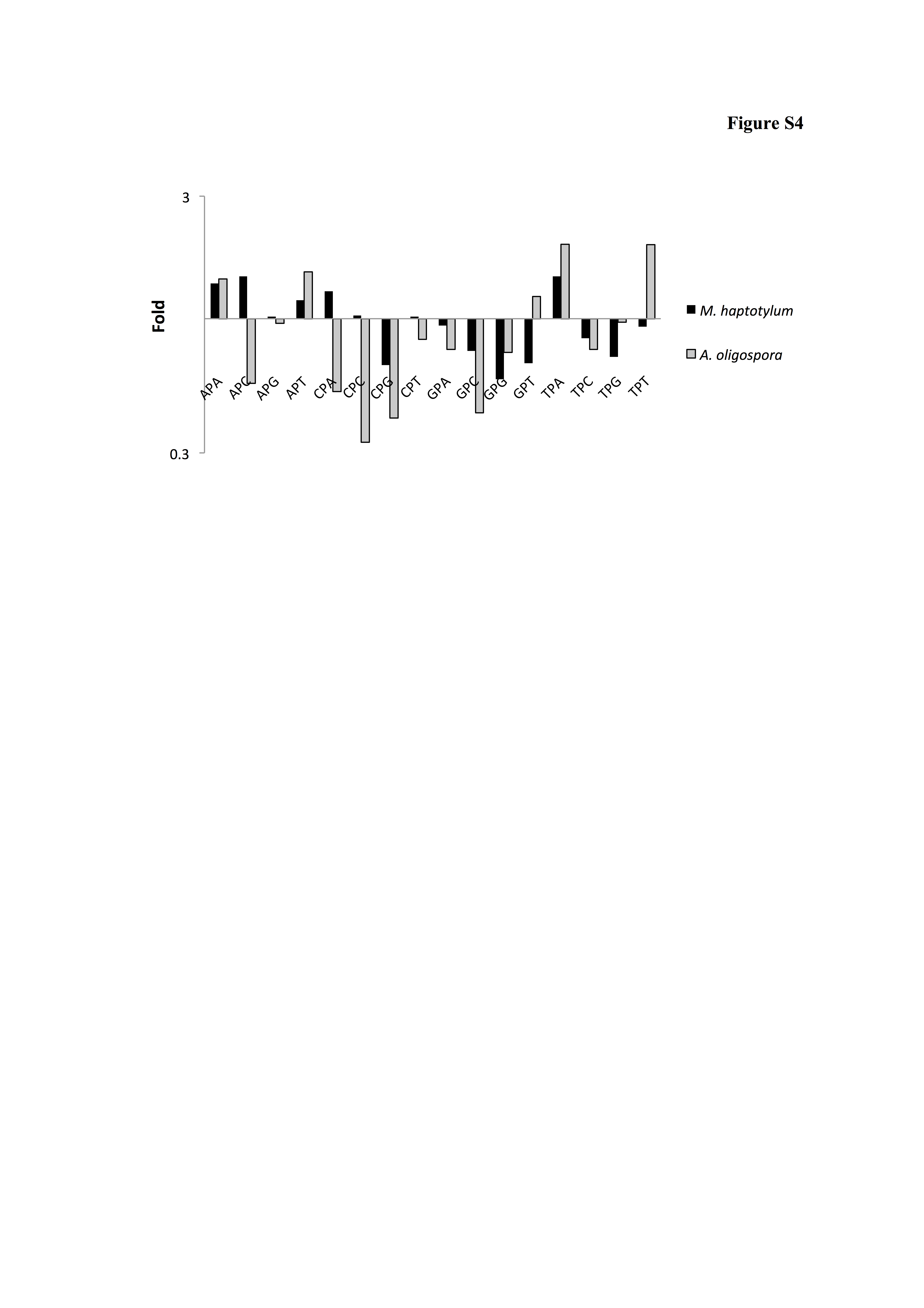

Supplement: Figure S4 — Fold changes in dinucleotide abundance for all repeat families in M. haptotylum and A. oligospora compared with non-repetitive control sequences on a log10 scale. This confirms the expected pattern of CpA→TpA type RIP mutations: high TpA and low TpG abundance. The difference in CpA differs between the two species. (TIFF) [file pgen.1003909.s005.tif]

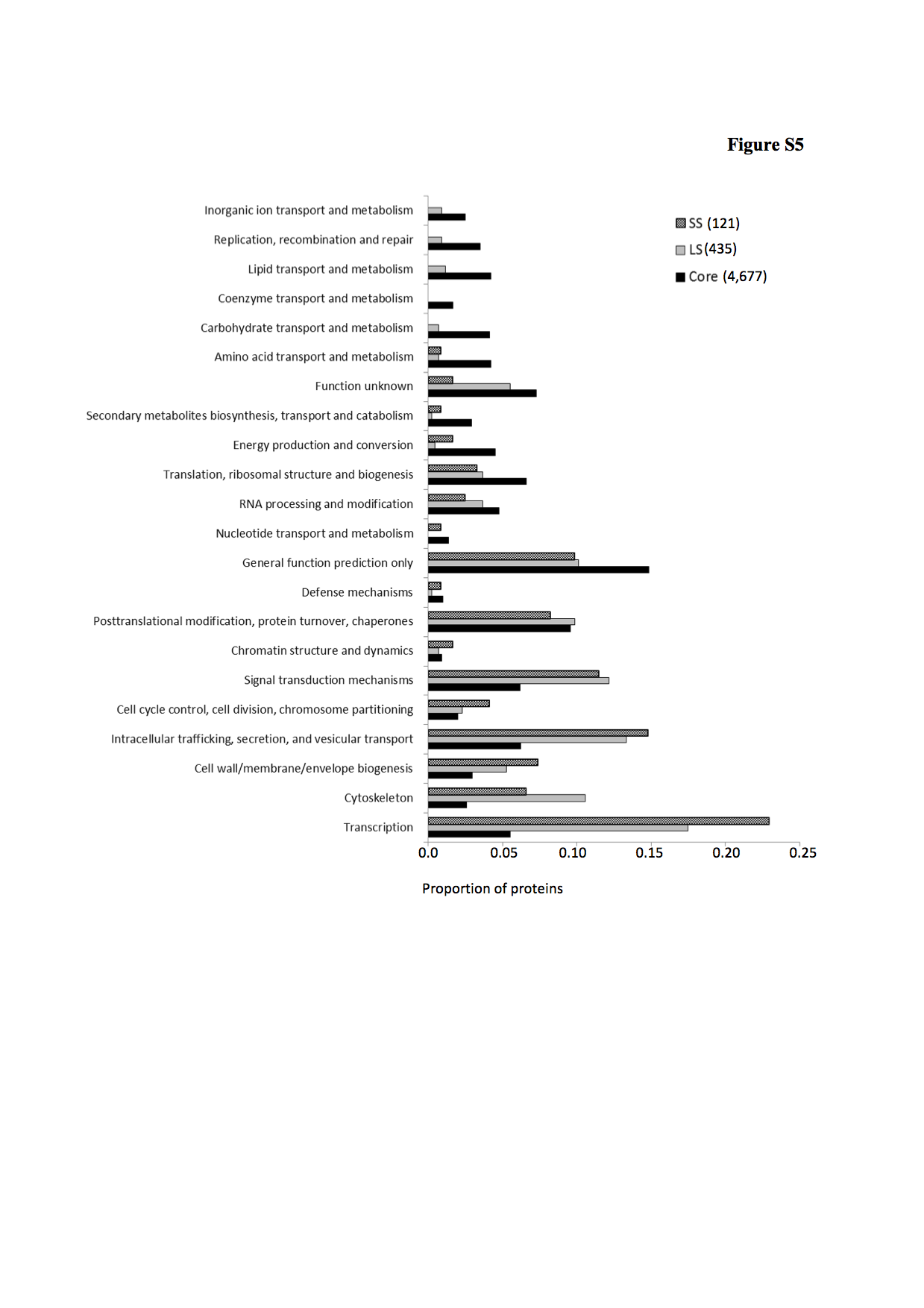

Supplement: Figure S5 — Functional classifications of core, lineage- and species-specific protein-coding genes in M. haptotylum. The proportions of proteins that have homologs in the the EuKaryotic Orthologous Groups (KOG) of proteins are shown. The total number of KOG proteins in the core, lineage-specific (LS) and species-specific (SS) categories are shown in parentheses. Protein sequences with multiple hits were excluded from the analysis. (TIFF) [file pgen.1003909.s006.tif]

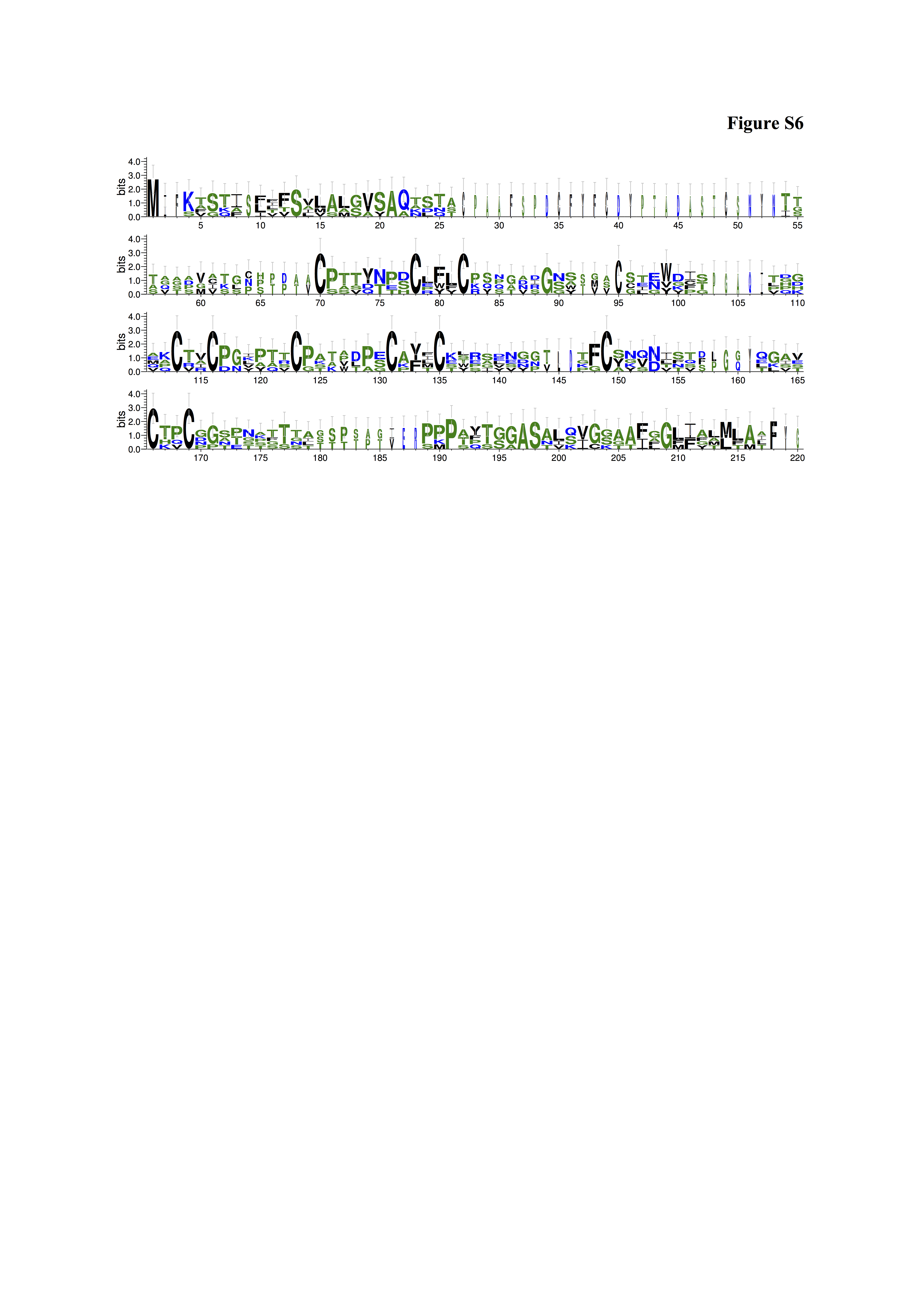

Supplement: Figure S6 — Amino acid representation of a cluster of five small secreted orphan proteins. The largest cluster containing only orphan SSPs was chosen (Figure 7). In the alignment, 12 cysteine residues were highly conserved while the rest of the positions were more variable. The signal peptide cleavage site was predicted between positions 20 and 21. (TIFF) [file pgen.1003909.s007.tif]
