# Supplementary material for: Genomic Mechanisms Accounting for the Adaptation to Parasitism in Nematode-Trapping Fungi
Source: PLoS Genet. 2013 Nov 14;9(11):e1003909. doi: 10.1371/journal.pgen.1003909 (PMC3828140; doi:10.1371/journal.pgen.1003909)
Supplement: Table S1 — Genome statistics of M. haptotylum. (DOCX) [file pgen.1003909.s008.docx]

**Table S1. Genome statistics of *M. haptotylum*.**

| General features | Values^a^ |
| --- | --- |
| Total number of reads assembled | Shotgun XLR70: 2.1×10^6^  Paired end: 1.0×10^6^ |
| Size (Mb) | 40.4 |
| GC content | 45.24 |
| Coverage | 28x |
| Number of scaffolds | 1279 |
| Mean scaffold size (bp) | 67,560 |
| N50 scaffold size (kb) | 194 |
| Largest scaffold size (kb) | 937 |
| Number of scaffolds >3 kb | 25 |
| Number of scaffolds >2 kb | 28 |
| Number of scaffolds >1 kb | 83 |
| Contigs | 1,543 |
| Largest contig (kb) | 818 |
| N50 contig size (kb) | 177 |
| Number of contigs >2 kb | 49 |
| Number of contigs >1 kb | 85 |
| Number of predicted gene models | 10,965 |
| Total number of exons | 36,272 |
| Average length of the exons (bp) | 469 |
| Number of single exon genes | 2,336 |
| Average length of introns (bp) | 108.60 |
| Total number of gaps | 34,178 |
| Total number of tRNA genes | 149 |

^a^Values were calculated using the Eval software [1]

1 Keibler E, Brent MR (2003) Eval: a software package for analysis of genome annotations. BMC Bioinformatics 4: 50.
