# Supplementary material for: Genomic Mechanisms Accounting for the Adaptation to Parasitism in Nematode-Trapping Fungi
Source: PLoS Genet. 2013 Nov 14;9(11):e1003909. doi: 10.1371/journal.pgen.1003909 (PMC3828140; doi:10.1371/journal.pgen.1003909)
Supplement: Table S2 — Mapping of transcriptome sequences against the genome assembly and gene models of M. haptotylum. (DOCX) [file pgen.1003909.s009.docx]

**Table S2. Mapping of transcriptome sequences against the genome assembly and gene models of *M. haptotylum*.**

| Data set |  | Mapping to genome | |  |  | Mapping to gene models | |  |  |
| --- | --- | --- | --- | --- | --- | --- | --- | --- | --- |
|  |  | Total number of sequences/reads | Number mapped to genome | | % mapped to genome | Total number of sequences | Number mapped to gene models | | % mapped to gene models |
| Sanger ESTs | Mycelium | 1,729 | 1,715 | | 99 | 1,729 | 1,187 | | 68 |
| Sanger ESTs | Knobs | 1,450 | 1,209 | | 83 | 1,450 | 455 | | 31 |
| 454 sequencing | Mycelium | 422,883 | 418,500 | | 99 | 11,221^a^ | 10,008 | | 90 |

^a^_The 454 pyrosequencing reads were assembled before mapping to the gene models._
