# Supplementary material for: Genomic Mechanisms Accounting for the Adaptation to Parasitism in Nematode-Trapping Fungi
Source: PLoS Genet. 2013 Nov 14;9(11):e1003909. doi: 10.1371/journal.pgen.1003909 (PMC3828140; doi:10.1371/journal.pgen.1003909)
Supplement: Table S3 — Fungal genomes analyzed in this study. (DOCX) [file pgen.1003909.s010.docx]

**Table S3. Fungal genomes analyzed in this study.**

| Species (strain) | Abbreviation | Genome size (Mb) | Protein-coding genes | NCBI /GenBank accessions | Release/ Update date | Lifestyle^a^ |
| --- | --- | --- | --- | --- | --- | --- |
| *Ashbya gossypii* (ATCC 10895) | AG | 9.2 | 4,726 | NC_005782 to 88 | 2004.03.04 | PPA |
| *Aspergillus niger* (CBS 513.88) | AN | 33.9 | 14,165 | Deposited at EMBL db – AM270980-AM270998 (DNA Supercontigs) and AM269948-AM270415 (DNA contigs) | 2007 | SAP |
| *Candida albicans* (SC5314) | CA | 14.3 | 6,094 | AAFO00000000 | 2002.5.24 (Assembly 19) | APA |
| *Fusarium graminearum* | FG | 36.1 | 13,321 | DV998659-DV998726 and DW005257-DW005273 | 2007 | PPA |
| *Magnaporthe grisea* (70_15) | MGR | 37.8 | 11,109 | GenBank/EMBL/DDBJ - AACU01000000 | 2002-09-17 | PPA |
| *Malassezia globosa* (CBS 7966) | MGL | 9.0 | 4,285 | AAY000000049 | 2007-11-03 | APA |
| *Saccharomyces cerevisiae* (S288C) | SC | 12.07 | 6,575 | NC_001133 – NC_001224 | 2006 | SAP |
| *Stagonospora nodorum* (SN15) | SN | 37.2 | 16,597 | AAGl0100000 | 5/13/2005 and 5/6/2011 | PPA |
| *Trichoderma reesei* (QM9414) | TR | 34.0 | 9,129 | AAIL01000000 | 2003.7.18 | SAP |
| *Coccidioides immitis* (RS.11-JUN-2007) | CI | 28.9 | 10,335 | AAEC02000000 | 2004.3.11 | APA |
| *Emericella nidulans* (FGSC_A4) | WN | 30.1 | 9,410 | AACD01000000 | 2005 | SAP |
| *Neurospora crassa* (OR74A.10-SEP-2007) | NC | 40.0 | 9,841 | AABX01000000 | 2005.2.17 | SAP |
| *Aspergillus fumigatus* (Af293. 12 May 2007) | AF | 29.4 | 9,630 | NC_007194 to NC_007201 | 2004.3.17 | APA |
| *Schizosaccharomyces pombe* (972h 31 Mar 2007) | SP | 12.5 | 5,032 | AL355920, AL355921, AL391034 and AL391016 | 2007 | SAP |
| *Podospora anserina* (DSM 980.24 Jun 2008) | PA | 35.5-36 | 10,118 |  | 2008 | SAP |
| *Tuber melanospora* (v1.0) | TM | 125 | 12,826 | CABJ01000001-CABJ01004455 and FN429986-FN430383 | 2010 | SYM |
| *Arthroderma benhamiae* (CBS 112371) | AB | 22.5 | 7,980 | ABSU00000000 | 2010 | APA |
| *Cryptococcus neoformans var neoformans* (JEC21.20-Apr-2007) | CN | 19 | 6,475 | NC_006670 to NC_006794 | 2005-01-13 | APA |
| *Metarhizium anisopliae* | MAA | 39.04 | 10,583 | ANDJ00000000 | 2010 | APA |
| *Metarhizium acridium* | MAC | 38.05 | 9,849 | ANDI00000000 | 2010 | APA |
| *Monacrosporium haptotylum* (CBS 200.50) | MH | 40.4 | 10,965 |  |  | APA |
| *Arthrobotrys oligospora* (ATCC 24927) | AO | 40.07 | 11,469 | ADOT00000000 | 2011 | APA |
| *Neosartorya fischeri (NRRL 181)* | NF | 33.2 | 10,406 | AAKE00000000.3 | 23-02-2009 | SAP |

^a^PPA, plant pathogen; SAP, saprophyte; APA, animal pathogen; SYM, symbiont (on plants)
