# Supplementary material for: Genomic Mechanisms Accounting for the Adaptation to Parasitism in Nematode-Trapping Fungi
Source: PLoS Genet. 2013 Nov 14;9(11):e1003909. doi: 10.1371/journal.pgen.1003909 (PMC3828140; doi:10.1371/journal.pgen.1003909)
Supplement: Table S4 — tRNA genes along with their respective anticodons present in M. haptotylum. (DOCX) [file pgen.1003909.s011.docx]

**Table S4. tRNA genes along with their respective anticodons present in *M. haptotylum*.**^a^

| Amino acid | Total counts |  | Anticodons | | | |  | | |  |
| --- | --- | --- | --- | --- | --- | --- | --- | --- | --- | --- |
| tRNA-? (Lys\|Stop)(ctta) | 3 |  | |  | | CTTA:2 | | TCCA:1 | | |
| tRNA-Ala | 8 | AGC:3 | | GGC:2 | | CGC:2 | | TGC:1 | | |
| tRNA-Arg | 16 | ACG:3 | | GCG:0 | | CCG:1 | | TCG:3 | | |
|  |  |  | |  | | CCT:3 | | TCT:6 | | |
| tRNA-Asn | 6 | ATT:3 | | GTT:3 | |  | |  | | |
| tRNA-Asp | 8 | ATC:0 | | GTC:8 | |  | |  | | |
| tRNA-Cys | 4 | ACA:0 | | GCA:4 | |  | |  | | |
| tRNA-Gln | 5 |  | |  | | CTG:2 | | TTG:3 | | |
| tRNA-Glu | 8 |  | |  | | CTC:3 | | TTC:4 | | |
|  |  |  | |  | | CTC(C):1 | |  | | |
| tRNA-Gly | 6 | ACC:0 | | GCC:3 | | CCC:0 | | TCC:3 | | |
| tRNA-His | 4 | ATG:0 | | GTG:4 | |  | |  | | |
| tRNA-Ile | 7 | AAT:5 | | GAT:1 | |  | | TAT:1 | | |
| tRNA-Leu | 12 | AAG:0 | | GAG:0 | | CAG:1 | | TAG:7 | | |
|  |  |  | |  | | CAA:2 | | TAA:2 | | |
| tRNA-Lys | 5 |  | |  | | CTT:2 | | TTT:3 | | |
| tRNA-Met | 8 |  | |  | | CAT:8 | |  | | |
| tRNA-Phe | 1 | AAA:0 | | GAA:1 | |  | |  | | |
| tRNA-Pro | 6 | AGG:2 | | GGG:0 | | CGG:0 | | TGG:4 | | |
| tRNA-Ser | 13 | AGA:4 | | GGA:1 | | CGA:1 | | TGA:3 | | |
|  |  |  | | GCT:4 | |  | |  | | |
| tRNA-Thr | 11 | AGT:5 | | GGT:0 | | CGT:1 | | TGT:5 | | |
| tRNA-Trp | 1 |  | |  | | CCA:1 | |  | | |
| tRNA-Tyr | 3 | ATA:0 | | GTA:3 | |  | |  | | |
| tRNA-Val | 13 | AAC:6 | | GAC:0 | | CAC:5 | | TAC:2 | | |
| tRNA-seC | 1 |  | |  | |  | | TCA:1 | | |
| Total | 149 |  | | |  | |  | |  | |

^a^Values were predicted using the Aragorn program [76].
