# Supplementary material for: Genomic Mechanisms Accounting for the Adaptation to Parasitism in Nematode-Trapping Fungi
Source: PLoS Genet. 2013 Nov 14;9(11):e1003909. doi: 10.1371/journal.pgen.1003909 (PMC3828140; doi:10.1371/journal.pgen.1003909)
Supplement: Table S5 — Families of transposable elements identified in the genomes of M. haptotylum and A. oligospora. (DOCX) [file pgen.1003909.s012.docx]

**Table S5. Families of transposable elements identified in the genomes of *M. haptotylum* and *A. oligospora.***

| **Type** | ***M. haptotylum*** | ***A. oligospora*** |
| --- | --- | --- |
| **Retrotransposons (Class I)** |  |  |
| DDE_1 | 1 | 3 |
| Gypsy | 201 | 60 |
| LINE | 3 | 38 |
| ltr_Roo | 0 | 1 |
| TY1_Copia | 18 | 12 |
| Total Class I | 223 | 114 |
| **DNA transposons (Class II)** |  |  |
| Cacti | 30 | 23 |
| hAT | 8 | 2 |
| helitronORF | 2 | 2 |
| Mariner | 26 | 3 |
| MuDR_A_B | 7 | 5 |
| P_element | 1 | 1 |
| Mariner_ant1 | 14 | 0 |
| Total Class II | 88 | 36 |
| ISC1316 | 3 | 2 |
| **Total TE** | 314 | 152 |
