# Supplementary material for: Genomic Mechanisms Accounting for the Adaptation to Parasitism in Nematode-Trapping Fungi
Source: PLoS Genet. 2013 Nov 14;9(11):e1003909. doi: 10.1371/journal.pgen.1003909 (PMC3828140; doi:10.1371/journal.pgen.1003909)
Supplement: Table S6 — Virulence-associated proteins in the genomes of M. haptotylum and A. oligospora. (DOCX) [file pgen.1003909.s013.docx]

**Table S6. Virulence associated proteins in the genomes of *M. haptotylum* and *A. oligospora****.*

| PHI-base ID | PHI-base gene | Description (effect on virulence) | *M. haptotylum*  Number of gene models ^a^ | *A. oligospora*  Number of gene models ^a^ |
| --- | --- | --- | --- | --- |
| **Transporter** |  |  |  |  |
| PHI:538 | FRT1 | Fructose transporter (unaffected) | 31 | 32 |
| PHI:544 | BCMFS1 | Transporter (unaffected) | 15 | 16 |
| PHI:1018 | ABC3 | Transporter (loss) | 12 | 12 |
| **Signaling** |  |  |  |  |
| PHI:188 | SNF1 | Protein kinase (reduced) | 13 | 11 |
| PHI:339 | CLPT1 | Signal transduction (reduced) | 12 | 14 |
| **Oxidation** |  |  |  |  |
| PHI:438 | BcBOT1 | P450 oxidation (reduced) | 25 | 19 |
| PHI:1100 | Mdh1 | Oxidation-reduction (unaffected) | 15 | 13 |
| **Transcription regulation** |  |  |  |  |
| PHI:211 | CaTUP1 | Transcription regulator (reduced) | 45 | 53 |
| PHI:423 | VAD1 | Transcription regulator (reduced) | 23 | 25 |
| PHI:482 | LAEA | Pathogenesis regulator (reduced) | 11 | 11 |
| **Metabolism** |  |  |  |  |
| PHI:541 | LIP1 | Lipase (unaffected) | 21 | 16 |
| PHI:226 | PEX6 | Fatty acid metabolism (lost) | 15 | 15 |
| PHI:569 | XYL3 | Xylanase (unaffected) | 15 | 19 |
| PHI:144 | CHT42 | Chitinase (reduced) | 5 | 11 |
| **Other** |  |  |  |  |
| PHI:256 | GAS1 | Host entry (reduced) | 25 | 21 |
| PHI:901 | um01886 | Unknown (unaffected) | 11 | 3 |
| PHI:184 | RBT4 | Pathogenesis (reduced) | 10 | 8 |

^a^Shown is the number of gene models matching proteins in the pathogen–host interaction (PHI-base) protein database containing a collection of experimentally verified genes from different fungi and bacteria. Only matches having at least ten gene models in either *M. haptotylum* or *A. oligospora* are shown.
