# Supplementary material for: Genomic Mechanisms Accounting for the Adaptation to Parasitism in Nematode-Trapping Fungi
Source: PLoS Genet. 2013 Nov 14;9(11):e1003909. doi: 10.1371/journal.pgen.1003909 (PMC3828140; doi:10.1371/journal.pgen.1003909)
Supplement: Table S7 — Major protein families of M. haptotylum implicated in pathogen–host interactions. (DOCX) [file pgen.1003909.s014.docx]

**Table S7. Major protein families of *M. haptotylum* implicated in pathogen–host interactions**^a^

| Domain ID | Domain name | Description | Number of proteins |
| --- | --- | --- | --- |
| PF00069 | Pkinase | Protein kinase family | 83 |
| PF00400 | WD40 | Beta-transducin repeat | 60 |
| PF07690 | MFS_1 | Major facilitator family | 40 |
| PF00734 | CBM_1 | Carbohydrate-binding module | 37 |
| PF00067 | p450 | Cytochrome P450 | 31 |
| PF00083 | Sugar_tr | Sugar and other transporter | 31 |
| PF00106 | adh_short | Short-chain dehydrogenase | 30 |
| PF00005 | ABC_tran | ABC transporter-like | 26 |
| PF11327 | DUF3129 (gas1) | Protein of unknown function | 26 |
| PF00026 | Asp | Aspartate protease | 25 |
| PF00071 | Ras | Ras family (small GTPases) | 24 |
| PF00271 | Helicase_C | Helicase conserved C-terminal domain | 23 |
| PF00270 | DEAD | DEAD/DEAH box helicase | 23 |
| PF04082 | Fungal_trans | Fungal-specific transcription factor domain | 22 |
| PF00135 | COesterase | Carboxylesterase family | 21 |
| PF00072 | Response_reg | Response regulator receiver domain | 21 |
| PF00004 | AAA | Predicted AAA-ATPase | 21 |
| PF02518 | HATPase_c | Histidine kinase DNA gyrase B-and  HSP90-like ATPase | 20 |
| PF00295 | Glyco_hydro_28 | Glycosyl hydrolases family 28 | 19 |
| PF00122 | E1-E2_ATPase | E1-E2 ATPase | 18 |
| PF00702 | Hydrolase | Haloacid dehalogenase-like hydrolase | 18 |
| PF00107 | ADH_zinc_N | Zinc-binding dehydrogenase | 18 |
| PF00172 | Zn_clus | Fungal Zn (2) Cys (6) binuclear cluster domain | 17 |
| PF08240 | ADH_N | Alcohol dehydrogenase GroES-like domain | 17 |
| PF00096 | zf-C2H2 | Classical C2H2 and C2H6 zinc fingers | 16 |
| PF00512 | HisKA | His kinase A (phospho-acceptor) domain | 16 |
| PF00501 | AMP-binding | AMP-binding enzyme | 14 |
| PF00664 | ABC_membrane | ABC transporter transmembrane region | 14 |
| PF01565 | FAD_binding_4 | FAD binding domain | 14 |
| PF00450 | Peptidase_S10 | Serine carboxypeptidase | 13 |
| PF08031 | BBE | Berberine and berberine-like | 11 |
| PF00160 | Pro_isomerase | Cyclophilin type peptidyl-prolyl cis-  trans isomerase/CLD | 11 |
| PF01915 | Glyco_hydro_3_C | Glycosyl hydrolase family 3 C-terminal domain | 10 |
| PF08241 | Methyltransf_11 | Methyl transferase domain | 10 |
| PF00933 | Glyco_hydro_3 | Glycosyl hydrolase family 3N-terminal domain | 10 |
| PF00690 | Cation_ATPase_N | Cation transporter /ATPase, N-terminus | 10 |
| PF00149 | Metallophos | Calcineurin-like phosphoesterase | 10 |
| PF00188 | CAP | Cysteine-rich secretory protein | 10 |

^a^Shown are the most abundant Pfam domains found among the proteins of *M. haptotylum* matching proteins in the PHI database (c.f. Table S9).
