# Supplementary material for: Genomic Mechanisms Accounting for the Adaptation to Parasitism in Nematode-Trapping Fungi
Source: PLoS Genet. 2013 Nov 14;9(11):e1003909. doi: 10.1371/journal.pgen.1003909 (PMC3828140; doi:10.1371/journal.pgen.1003909)
Supplement: Table S8 — Expression of expanded orthoMCL gene families in M. haptotylum that were also expanded in plant pathogenic, insect pathogenic and animal (i.e. human) pathogenic fungi. (DOCX) [file pgen.1003909.s015.docx]

**Table S8. Expression of expanded orthoMCL gene families in *M. haptotylum* that were also expanded in plant pathogenic, insect pathogenic and animal (i.e. human) pathogenic fungi.**^a^

| **Gene_id** | **Category** | **OrthoMCL** | **PHI-base** | **Pfam domain** | **Secreted** | **Fold I/K ^b^** | **Fold K/M^c^** |
| --- | --- | --- | --- | --- | --- | --- | --- |
| **Upregulated infection hyphae** | |  |  |  |  |  |  |
| H072_8571 | Insect | asco_4661 | CaTUP1 | NACHT,Ank |  | 2,0 | 0,1 |
| H072_6491 | Plant | asco_7496 |  | CBM_1 |  | 3,0 | 0,8 |
| H072_4239 | Plant | asco_4726 |  | Sec61_beta |  | 2,8 | 1,0 |
| H072_9257 | Plant | asco_5146 |  | CMD |  | 2,3 | 1,5 |
| H072_5641 | Plant | asco_7466 | XYL3 | CBM_1 |  | 2,3 | 0,2 |
| H072_4752 | Plant | asco_6073 |  | MFS_1,MFS_1 |  | 2,2 | 0,8 |
| H072_9860 | Plant | asco_7469 |  |  | SSP | 2,2 | 0,2 |
| **Upregulated knobs** |  |  |  |  |  |  |  |
| H072_961 | Animal | asco_1506 |  | SKN1 |  | 1,1 | 4,3 |
| H072_10017 | Insect | asco_7729 |  | Glyoxalase |  | 0,2 | 5,7 |
| H072_55 | Insect | asco_7718 |  | YCII |  | 0,1 | 5,4 |
| H072_5845 | Insect | asco_5708 |  | dCMP_cyt_deam_1 |  | 0,8 | 3,0 |
| H072_6774 | Plant | asco_6325 |  | Methyltransf_11 |  | 0,2 | 4,8 |
| H072_8197 | Plant | asco_6792 |  |  |  | 0,8 | 3,1 |
| H072_3926 | Plant | asco_6943 |  | Epimerase |  | 0,7 | 2,1 |

^a^ Expanded gene families were identified using the CAFE tool, and the expansions shared between the nutritional types of fungi were identified using the F-test. The categories are shown in the heat map in Figure 3B. Only genes that were more than twofold upregulated are shown.

^b^ “Fold I/K” is the ratio of gene expression levels (RNAseq data) in the infecting hyphae (I) and knob (K).

^c^ ”Fold K/M” is the ratio value of gene expression levels (RNAseq data) in the knob (K) and mycelium (M). Data of the expression in the mycelium was obtained from [68].
