# Supplementary material for: Genomic Mechanisms Accounting for the Adaptation to Parasitism in Nematode-Trapping Fungi
Source: PLoS Genet. 2013 Nov 14;9(11):e1003909. doi: 10.1371/journal.pgen.1003909 (PMC3828140; doi:10.1371/journal.pgen.1003909)
Supplement: Table S9 — Gene families significantly expanded and contracted in nematode-trapping fungi in comparison with 19 other fungal species. (DOCX) [file pgen.1003909.s016.docx]

**Table S9. Protein domain families significantly expanded and contracted in nematode-trapping fungi in comparison with 19 other fungal species**.^a^

| **Pfam^b^** | **Description** | **P -value^c^** | **PHI^d^** | **MH** | **AO** | **MAA** | **MGR** | **AB** | **CI** | **TM** | **MGL** | **SP** | **SC** | **AG** | **NC** | **TR** | **AN** | **AF** | **WN** | **FG** | **PA** | **SN** | **CN** | **CA** |
| --- | --- | --- | --- | --- | --- | --- | --- | --- | --- | --- | --- | --- | --- | --- | --- | --- | --- | --- | --- | --- | --- | --- | --- | --- |
| **PF11327** | DUF3129 (gas1) | 0 | Φ | 33 | 33 | 7 | 8 | 0 | 2 | 1 | 0 | 0 | 0 | 0 | 2 | 0 | 0 | 1 | 0 | 4 | 2 | 4 | 0 | 0 |
| **PF00734** | CBM_1 (Cellulose-binding module) | 0 | Φ | 108 | 85 | 4 | 22 | 0 | 0 | 4 | 0 | 1 | 0 | 0 | 18 | 14 | 8 | 17 | 6 | 12 | 30 | 12 | 0 | 0 |
| **PF00646** | F-box domain (Protein-protein interaction) | 0 | Φ | 190 | 214 | 33 | 29 | 38 | 34 | 64 | 8 | 12 | 11 | 9 | 30 | 33 | 48 | 34 | 38 | 50 | 35 | 63 | 12 | 17 |
| PF10528 | GLEYA domain (fungal adhesins) | 0 |  | 28 | 4 | 5 | 1 | 0 | 0 | 0 | 0 | 5 | 0 | 1 | 0 | 2 | 0 | 0 | 1 | 3 | 0 | 1 | 0 | 0 |
| PF01048 | PNP_UDP_1 (Phosphorylase superfamily) | 5.10E-13 | Φ | 49 | 72 | 27 | 10 | 2 | 3 | 2 | 1 | 2 | 2 | 2 | 3 | 25 | 34 | 12 | 22 | 11 | 8 | 2 | 1 | 2 |
| **PF00082** | Peptidase_S8 ([Subtilisin](http://en.wikipedia.org/wiki/Subtilisin)-like [serine proteases](http://en.wikipedia.org/wiki/Serine_protease)) | 7.20E-13 | Φ | 59 | 52 | 55 | 30 | 18 | 18 | 7 | 3 | 4 | 4 | 3 | 10 | 23 | 15 | 9 | 7 | 32 | 25 | 18 | 3 | 5 |
| PF01822 | WSC (Carbohydrate binding) | 4.60E-10 | Φ | 33 | 16 | 16 | 13 | 4 | 4 | 6 | 0 | 0 | 3 | 3 | 11 | 9 | 4 | 5 | 4 | 14 | 18 | 11 | 7 | 3 |
| **PF00023** | Ankyrin repeat (Protein-protein interactions) | 7.10E-10 | Φ | 139 | 139 | 108 | 57 | 47 | 42 | 363 | 11 | 14 | 18 | 14 | 46 | 59 | 86 | 58 | 57 | 99 | 83 | 93 | 15 | 15 |
| PF00264 | Tyrosinase | 1.30E-09 | Φ | 29 | 16 | 8 | 17 | 1 | 3 | 4 | 0 | 0 | 0 | 0 | 8 | 3 | 6 | 3 | 10 | 15 | 14 | 16 | 0 | 0 |
| **PF02480** | Herpes_gE (Alphaherpesvirus glycoprotein) | 3.80E-09 |  | 27 | 13 | 8 | 11 | 1 | 5 | 9 | 1 | 3 | 3 | 2 | 6 | 3 | 5 | 6 | 3 | 7 | 13 | 14 | 3 | 1 |
| PF05729 | NACHT domain | 4.30E-09 | Φ | 76 | 105 | 43 | 25 | 7 | 5 | 199 | 1 | 0 | 0 | 0 | 11 | 32 | 37 | 16 | 19 | 57 | 46 | 36 | 2 | 0 |
| PF00295 | Glycoside hydrolase family 28 | 4.20E-08 | Φ | 20 | 7 | 1 | 3 | 0 | 0 | 2 | 0 | 0 | 1 | 1 | 2 | 4 | 19 | 12 | 9 | 6 | 0 | 4 | 1 | 0 |
| **PF01456** | Mucin-like glycoprotein | 7.30E-08 | Φ | 16 | 8 | 3 | 2 | 1 | 3 | 2 | 0 | 0 | 0 | 0 | 2 | 0 | 2 | 4 | 5 | 8 | 3 | 1 | 0 | 2 |
| PF03443 | Glycoside hydrolase family 61 | 3.60E-06 | Φ | 28 | 26 | 3 | 25 | 1 | 1 | 4 | 0 | 0 | 0 | 0 | 14 | 4 | 7 | 8 | 9 | 14 | 33 | 31 | 1 | 0 |
| **PF00413** | Peptidase M10 (Metallopeptidase) | 3.70E-06 |  | 9 | 7 | 5 | 0 | 0 | 0 | 0 | 0 | 0 | 0 | 0 | 0 | 0 | 0 | 0 | 1 | 2 | 1 | 1 | 0 | 0 |
| **PF00026** | Aspartyl protease | 0.000006 | Φ | 38 | 36 | 33 | 20 | 9 | 6 | 4 | 18 | 2 | 9 | 7 | 19 | 18 | 12 | 7 | 10 | 21 | 26 | 21 | 9 | 14 |
| **PF08693** | SKG6 (Transmembrane alpha-helix domain) | 1.30E-05 | Φ | 32 | 29 | 15 | 12 | 10 | 7 | 9 | 0 | 2 | 8 | 2 | 14 | 13 | 14 | 11 | 19 | 27 | 19 | 19 | 5 | 7 |
| **PF00651** | BTB/POZ domain (Protein-protein interaction) | 2.10E-05 | Φ | 36 | 58 | 16 | 14 | 9 | 14 | 12 | 2 | 3 | 1 | 1 | 17 | 14 | 12 | 12 | 10 | 27 | 11 | 57 | 4 | 3 |
| **PF04885** | Stig 1 (Cysteine rich plant protein) | 2.40E-05 | Φ | 6 | 5 | 0 | 0 | 0 | 0 | 0 | 0 | 0 | 0 | 0 | 0 | 0 | 0 | 1 | 0 | 1 | 0 | 0 | 0 | 0 |
| PF00622 | SPRY (Unknown function) | 3.90E-05 | Φ | 17 | 15 | 9 | 3 | 3 | 3 | 8 | 2 | 3 | 4 | 3 | 3 | 4 | 3 | 3 | 4 | 5 | 5 | 6 | 3 | 3 |
| **PF05730** | CFEM (Fungal-specific cysteine rich domain) | 0.00012 |  | 26 | 19 | 19 | 24 | 5 | 1 | 6 | 0 | 0 | 2 | 1 | 14 | 18 | 8 | 6 | 6 | 23 | 18 | 16 | 2 | 8 |
| PF00652 | Ricin type lectin domain | 0.0002 |  | 8 | 7 | 1 | 2 | 0 | 0 | 0 | 0 | 0 | 0 | 0 | 1 | 3 | 1 | 1 | 0 | 2 | 1 | 1 | 5 | 0 |
| PF01095 | Pectine esterase | 0.00025 | Φ | 8 | 5 | 0 | 1 | 0 | 1 | 1 | 0 | 0 | 0 | 0 | 1 | 0 | 2 | 4 | 3 | 3 | 1 | 4 | 0 | 0 |
| PF00931 | NB-ARC domain | 0.00034 |  | 17 | 16 | 12 | 7 | 5 | 6 | 5 | 0 | 0 | 0 | 0 | 3 | 4 | 9 | 6 | 12 | 15 | 9 | 5 | 0 | 0 |
| PF00544 | Pectate lyase | 0.00035 | Φ | 10 | 7 | 0 | 2 | 0 | 0 | 2 | 0 | 0 | 0 | 0 | 1 | 0 | 4 | 5 | 7 | 9 | 3 | 4 | 0 | 0 |
| PF00078 | Reverse transcriptase | 0 | Φ | 8 | 7 | 21 | 133 | 1 | 58 | 732 | 1 | 11 | 3 | 2 | 2 | 0 | 11 | 2 | 30 | 2 | 2 | 5 | 13 | 7 |
| PF00665 | Rve (Integrase core domain) | 2.30E-13 | Φ | 2 | 2 | 17 | 73 | 0 | 31 | 480 | 1 | 10 | 0 | 0 | 0 | 0 | 13 | 0 | 20 | 2 | 1 | 0 | 4 | 5 |
| PF00692 | dUTPase | 2.20E-09 |  | 1 | 1 | 1 | 1 | 2 | 1 | 404 | 1 | 1 | 1 | 1 | 1 | 1 | 2 | 3 | 1 | 1 | 1 | 1 | 2 | 1 |
| PF07690 | MFS_1 (Major Facilitator Superfamily) | 1.50E-08 | Φ | 97 | 96 | 221 | 184 | 132 | 117 | 66 | 25 | 52 | 51 | 32 | 108 | 166 | 306 | 223 | 271 | 254 | 155 | 272 | 135 | 68 |
| PF03184 | DDE superfamily endonuclease | 1.10E-07 |  | 0 | 0 | 8 | 162 | 0 | 20 | 51 | 0 | 3 | 1 | 1 | 1 | 0 | 11 | 0 | 21 | 4 | 0 | 4 | 2 | 8 |
| **PF06985** | HET (Heterokaryon in compatibility protein) | 2.20E-06 |  | 11 | 10 | 37 | 41 | 4 | 2 | 2 | 0 | 0 | 0 | 0 | 50 | 44 | 30 | 8 | 12 | 94 | 129 | 172 | 0 | 0 |
| PF07727 | RVT_2 Reverse transcriptase (RNA-dependent DNA polymerase | 8.40E-06 |  | 0 | 0 | 13 | 3 | 1 | 47 | 134 | 0 | 0 | 0 | 0 | 0 | 0 | 13 | 0 | 3 | 0 | 0 | 2 | 0 | 1 |
| PF00385 | Chromo (Chromatin Organisation Modifier) domain | 0.00001 |  | 6 | 11 | 8 | 105 | 6 | 14 | 199 | 4 | 7 | 2 | 3 | 9 | 7 | 4 | 5 | 24 | 10 | 7 | 8 | 8 | 2 |
| **PF00096** | zf_H2C2 (Zinc finger motif) | 1.10E-05 | Φ | 1 | 2 | 2 | 31 | 1 | 6 | 196 | 0 | 10 | 1 | 1 | 0 | 1 | 1 | 1 | 1 | 1 | 2 | 1 | 1 | 1 |
| **PF00172** | Zn_clus (Zinc finger proteins) | 1.30E-05 | Φ | 92 | 95 | 164 | 118 | 51 | 88 | 62 | 5 | 30 | 55 | 41 | 99 | 208 | 308 | 224 | 234 | 264 | 133 | 141 | 79 | 81 |
| **PF04082** | Fungal_trans (Fungal-specific transcription factor domain) | 2.10E-05 | Φ | 70 | 75 | 155 | 92 | 63 | 65 | 51 | 3 | 26 | 29 | 16 | 69 | 147 | 265 | 168 | 206 | 213 | 97 | 137 | 64 | 32 |
| PF00550 | PP-binding (Phosphopantetheine attachment site) | 5.50E-05 | Φ | 10 | 10 | 63 | 43 | 35 | 21 | 8 | 7 | 4 | 2 | 2 | 15 | 28 | 71 | 38 | 56 | 38 | 29 | 39 | 4 | 3 |
| PF08284 | RVP_2 Retroviral aspartyl protease | 6.50E-05 | Φ | 2 | 0 | 2 | 38 | 0 | 1 | 216 | 0 | 0 | 0 | 0 | 0 | 0 | 0 | 0 | 6 | 0 | 0 | 0 | 1 | 0 |
| PF05225 | HTH_psq (helix-turn-helix structural motif) | 9.50E-05 |  | 0 | 0 | 0 | 125 | 0 | 8 | 6 | 0 | 0 | 0 | 1 | 3 | 0 | 6 | 0 | 19 | 0 | 0 | 0 | 0 | 4 |
| PF05663 | DUF809 (Unknown function) | 0.00014 |  | 0 | 0 | 0 | 0 | 0 | 0 | 165 | 0 | 0 | 0 | 0 | 0 | 0 | 0 | 0 | 0 | 0 | 0 | 0 | 0 | 0 |
| PF08240 | ADH_N (Catalytic domain of alcohol dehydrogenases) | 0.00063 | Φ | 24 | 23 | 57 | 52 | 23 | 27 | 15 | 12 | 10 | 20 | 9 | 31 | 49 | 101 | 56 | 69 | 65 | 43 | 70 | 26 | 23 |
| PF03221 | Transposase_Tc5 (DNA-binding domain) | 0.00091 |  | 1 | 2 | 3 | 111 | 1 | 5 | 10 | 0 | 3 | 1 | 2 | 4 | 3 | 4 | 1 | 4 | 4 | 2 | 3 | 0 | 8 |

^a^The protein domain families in were identified using local search of the Pfam database (cutoff <0.05). The counts of gene models in each family were used as input for statistical analysis of over- and under-represented proteins using the hypogeometric test. Abbreviations of fungal taxa are given in Table S3.

^b^Pfam families in bold were also found among the lineage-specific families (Table S11).

^c^Significance was tested using the hypogeometric distribution. Shown are all families that are significantly expanded or contracted (p<0.001) in nematode-trapping fungi in comparison with 19 other fungal species.

^d^The symbol Φ indicates that the Pfam family contains *M. haptotylum* proteins that matches proteins (BlastP, cutoff value <1E -10) in the pathogen–host interaction (PHI-base) database [29].
