# Supplementary material for: Genomic Mechanisms Accounting for the Adaptation to Parasitism in Nematode-Trapping Fungi
Source: PLoS Genet. 2013 Nov 14;9(11):e1003909. doi: 10.1371/journal.pgen.1003909 (PMC3828140; doi:10.1371/journal.pgen.1003909)
Supplement: Table S10 — Repetitive sequences identified in the genomes of M. haptotylum and A. oligospora. (DOCX) [file pgen.1003909.s017.docx]

**Table S10. Repetitive sequences identified in the genomes of *M. haptotylum* and *A. oligospora.***

| *M. haptotylum A. oligospora* | | | | | | |
| --- | --- | --- | --- | --- | --- | --- |
| Type | Number of elements | Length occupied (bp) | Genome coverage (%) | Number of elements | Length occupied (bp) | Genome coverage (%) |
| LINEs | 178 | 15,781 | 0.039 | 276 | 26,964 | 0.067 |
| LTR elements | 05 | 572 | 0.001 | 11 | 709 | 0.002 |
| DNA elements | 08 | 434 | 0.001 | 08 | 419 | 0.001 |
| All | 221 | 18,999 | 0.047 | 323 | 30,223 | 0.075 |
