# Supplementary material for: Genomic Mechanisms Accounting for the Adaptation to Parasitism in Nematode-Trapping Fungi
Source: PLoS Genet. 2013 Nov 14;9(11):e1003909. doi: 10.1371/journal.pgen.1003909 (PMC3828140; doi:10.1371/journal.pgen.1003909)
Supplement: Table S11 — The most abundant Pfam domains identified among the lineage- and species-specific proteins of the nematode-trapping fungi M. haptotylum and A. oligospora. (DOCX) [file pgen.1003909.s018.docx]

**Table S11. The most abundant Pfam domains identified among the lineage-specific (LS) and species-specific (SS) proteins of the nematode-trapping fungi *M. haptotylum* and *A. oligospora*. Shown are families with at least six members.**

| Pfam^a^ | Description | PHI^b^ | *M. haptotylum* | |  |  | *A. oligospora* | |  |  |
| --- | --- | --- | --- | --- | --- | --- | --- | --- | --- | --- |
|  |  |  | SS | LS | | Core | S | LS | | Core |
| **PF00646** | F-box domain (Protein-protein interaction) | Φ | 53 | 111 | | 26 | 68 | 125 | | 21 |
| **PF00651** | BTB/POZ domain (Protein-protein interaction) | Φ | 6 | 22 | | 8 | 19 | 34 | | 5 |
| **PF00023** | Ankyrin repeat (Protein-protein interaction) | Φ | 9 | 21 | | 109 | 3 | 21 | | 115 |
| **PF05730** | CFEM (Fungal-specific cysteine rich domain) |  | 7 | 18 | | 1 | 1 | 16 | | 2 |
| **PF00734** | CBM_1 (Cellulose-binding module) | Φ | 5 | 15 | | 88 | 5 | 13 | | 67 |
| **PF08693** | SKG6 (Transmembrane alpha-helix domain) | Φ | 9 | 13 | | 10 | 3 | 14 | | 12 |
| PF00096 | zf-C2H2 (Zinc finger motif) | Φ | 3 | 13 | | 28 | 7 | 15 | | 29 |
| **PF02480** | Herpes_gE (alphaherpesvirus glycoprotein) |  | 11 | 10 | | 6 | 6 | 4 | | 3 |
| **PF00413** | Peptidase M10 (Metallopeptidase) |  | 0 | 9 | | 0 | 1 | 4 | | 2 |
| PF01284 | MARVEL (Membrane-associating domain) |  | 0 | 9 | | 7 | 0 | 10 | | 9 |
| **PF00082** | Peptidase S8 (Subtilisin-like serine proteases) | Φ | 1 | 9 | | 49 | 0 | 12 | | 40 |
| **PF00026** | Asp (Aspartyl protease) | Φ | 1 | 7 | | 30 | 2 | 11 | | 23 |
| PF10342 | Drmip_Hesp (Ser-Thr-rich glycosyl-phosphatidyl-inositol-anchored membrane family) |  | 2 | 6 | | 2 | 1 | 6 | | 1 |
| PF00069 | Pkinase (Protein kinase domain) | Φ | 0 | 6 | | 116 | 5 | 11 | | 109 |
| PF00172 | Zn_clus (Zinc finger proteins) | Φ | 5 | 6 | | 81 | 0 | 10 | | 85 |
| PF04478 | Mid2 (Mid2 like cell wall stress sensor) |  | 0 | 5 | | 4 | 3 | 6 | | 4 |
| PF05808 | Podoplanin |  | 3 | 4 | | 0 | 0 | 8 | | 2 |
| **PF11327** | DUF3129 (gas1) | Φ | 1 | 4 | | 28 | 0 | 7 | | 26 |
| PF01544 | CorA (Metal ion transporter) |  | 3 | 4 | | 18 | 6 | 3 | | 19 |
| PF04156 | IncA | Φ | 9 | 4 | | 13 | 5 | 5 | | 13 |

^a^Pfam families in bold text were also identified among the most expanded gene families of *M. haptotylum* (Figure 3).

^b^The symbol Φ indicates that the Pfam family contains *M. haptotylum* proteins that matches proteins in the pathogen–host interaction (PHI-base) database.
